# Does the addition of hip strengthening exercises improve outcomes following total knee arthroplasty? A study protocol for a randomized trial

**DOI:** 10.1186/s12891-016-1104-x

**Published:** 2016-06-13

**Authors:** Margaret B. Schache, Jodie A. McClelland, Kate E. Webster

**Affiliations:** School of Allied Health, La Trobe University, Melbourne, Australia; Physiotherapy Department, Donvale Rehabilitation Hospital, Ramsay Health Care, 1119 Doncaster Rd, Donvale, Melbourne 3111 Australia

**Keywords:** Total knee arthroplasty, Hip strengthening, Randomized controlled trial, Exercise, Rehabilitation

## Abstract

**Background:**

Total knee arthroplasty (TKA) is effective in reducing pain and improving function for end-stage knee osteoarthritis. However, muscle weakness and functional limitations persist despite assistance from post-operative rehabilitation programs that traditionally focus on quadriceps strengthening and range of movement exercises. Hip abductor muscle weakness is evident in knee osteoarthritis and hip muscle strengthening reduces knee pain in this group. Following TKA, people with weak hip abductor strength perform more poorly on measures of physical function. However, very little is known of the effectiveness of including hip abductor strengthening exercises in post-operative rehabilitation. The aim of this trial is to compare the effects of targeted hip abductor strengthening to those of traditional care in a TKA rehabilitation program on muscle strength, patient reported outcomes and functional performance measures.

**Methods/design:**

This protocol describes a single-blinded randomized controlled trial, where 104 participants referred for inpatient rehabilitation following TKA will be recruited. Participants will be randomized using computer-generated numbers to one of two groups: usual care or usual care with additional hip strengthening exercises. Participants will attend physiotherapy daily during their inpatient length of stay, and will then attend between six and eight physiotherapy sessions as an outpatient. Primary outcomes are isometric hip abductor strength and the Knee Injury and Osteoarthritis Outcome Score (KOOS). Secondary outcomes are stair climb test, 6 min walk test, timed up and go, 40 m fast-paced walk test, 30 second chair stand test, isometric quadriceps strength, Lower Extremity Functional Scale (LEFS) and SF-12. Outcome measures will be recorded at baseline (admission to inpatient rehabilitation), and then 3 weeks, 6 weeks and 6 months post admission to rehabilitation.

**Discussion:**

The findings of this study will determine whether the addition of targeted hip strengthening to usual care rehabilitation improves physical performance and patient reported outcomes following TKA when compared to usual care rehabilitation. This will then determine whether targeted hip strengthening exercises should be included in traditional rehabilitation programs to improve the outcomes following total knee arthroplasty.

**Trial registration:**

The trial protocol was registered with the Australian Clinical Trial Registry (ACTRN12615000863538) on 18 August 2015.

**Electronic supplementary material:**

The online version of this article (doi:10.1186/s12891-016-1104-x) contains supplementary material, which is available to authorized users.

## Background

End-stage knee osteoarthritis (OA) is a significant health issue worldwide resulting in severe pain and disability [1, 2]. Total knee arthoplasty (TKA) leads to significant improvements in pain and the performance of functional activities such as walking for patients with end-stage knee OA [3]. Improvements in pain and functional performance are achieved with assistance from rehabilitation programs [3–6]. Traditional rehabilitation programs typically focus on improving knee strength and range of movement, and improving gait and stair climbing [7–10]. For most programs, emphasis is placed on achieving optimal knee range of movement and improving quadriceps muscle strength [11].

Despite completing traditional rehabilitation programs, functional limitations persist in patients following TKA compared to healthy age-matched individuals. Following TKA, people have reduced walking speed, and report difficulty climbing stairs and rising from a chair [12, 13]. They also perform more poorly on tests of Timed Up and Go (TUG) and stair climbing times than healthy adults [3, 13]. The persistence of functional limitations demonstrates that there is a need to expand on the focus of current rehabilitation practices to restore the function of patients following TKA to the levels of healthy adults.

Prior to surgery, patients with end-stage knee OA demonstrate reduced hip abductor strength [14]. They exhibit altered gait patterns that may be an attempt to avoid knee pain, to minimize forces through the affected cartilage, or to reduce the sensation of the knee giving way [15]. Consequently, patients with severe knee OA walk with decreased gait speed, reduced stride length, and increased time in double limb support [16]. These factors combined with reduced activity may result in lowered activation and diminished strength of the hip abductors over a period of time [17]. There has been a small quantity of research with promising findings that targeted hip strengthening programs in patients with end stage knee OA may lead to improvement in symptoms and quality of life [18, 19].

Although often ignored in post-operative rehabilitation programs, there is sufficient evidence that the hip abductor weakness present prior to surgery continues after TKA and is not improved with current rehabilitation interventions [20, 21]. Post-operative pain, reduced demands on the operated limb in the early post-operative period and habitual gait patterns are also likely to contribute to further reductions in hip abductor strength. Following TKA, there is a correlation between hip abductor strength and functional outcomes [20, 21]. Hip abductor strength contributes to physical function such as turning whilst walking and rising from a chair in people with unilateral TKA. There is a correlation between stronger hip abductor muscles and faster times for TUG and stair climb tests [20, 21]. Unless rehabilitation programs specifically include hip abductor strengthening exercises, it is unlikely that the hip abductors will return to normal levels of strength, contributing to ongoing difficulties in activities of daily living such as walking and stair climbing. Given the improvement in strength seen in pre-operative patients, it is likely that similar rehabilitation efforts will yield improvements in function post-operatively. Despite this, no study has investigated the effects of hip abductor strengthening on improving strength or function following TKA.

The aim of this randomized controlled trial (RCT) is to compare the effects of targeted hip abductor strengthening with those of traditional care in a TKA rehabilitation program on muscle strength, patient reported outcomes and functional performance measures.

## Methods/design

### Trial design

**Fig. 1:**
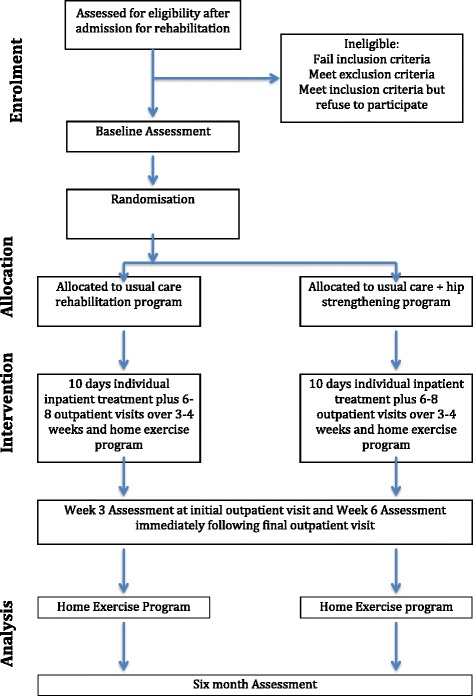
Flow diagram of study protocol

The study design is a single-blinded randomized controlled trial (Fig. 1) adhering to Standard Protocol Items: Recommendation for Interventional Trials (SPIRIT) guidelines [22]. Outcomes will be measured at admission to inpatient rehabilitation, and at 3 weeks, 6 weeks and 6 months following the commencement of rehabilitation. These time points were chosen to adequately measure the rate of improvement in all outcome measures.

The trial protocol was registered with the Australian New Zealand Clinical Trials Registry (ANZCTR number: 12615000863538) on 18 August 2015. Ethical approval was granted by La Trobe University Faculty Human Ethics Committee (FHEC 14/256). Reporting of the RCT will follow the CONSORT guidelines for randomized trials of nonpharmacologic treatment (Fig. 1) [23].

### Participants

A sample of 104 males and females with primary unilateral TKA will be recruited between October 2015 and March 2017 from a 90 bed private rehabilitation hospital that treats patients following joint replacements, multi-trauma, stroke, cardiac surgery and general deconditioning.

Participants will be eligible if they are aged 50 years or older, have had a primary unilateral TKA for end-stage knee osteoarthritis in the previous two weeks and are admitted for inpatient rehabilitation followed by outpatient rehabilitation.

Participants will be excluded in the presence of unstable medical conditions preventing the patient from participating in the rehabilitation programs, history of ipsilateral hip replacement, ipsilateral hip osteoarthritis or lateral hip pain, neurological or any other conditions affecting strength or function of lower limbs. Eligibility of prospective patients will be identified on their admission to inpatient rehabilitation from the acute hospitals by the treating physiotherapists.

### Randomisation and allocation concealment

Eligible patients will be identified by the treating physiotherapist. The treating physiotherapist will inform the patient of the study, give them the patient information sheet and arrange for a meeting with the primary researcher. The primary researcher will discuss any questions with the patient and gain informed consent of willing participants. Eligible patients will be randomized by a computer-generated list of random numbers to usual care or usual care plus hip strengthening. An independent person, not involved in delivering the intervention or testing, will manage the allocation list and numbered opaque envelopes containing the allocated group of each participant. Only the treating physiotherapist will be informed of the group allocation of the participants. The allocation list will not be available to the researchers and the participants will not be informed which group they are in. The participants will be informed that there are two different rehabilitation programs but will not be told that one program is specifically targeting hip strengthening exercises. This is to prevent potential bias from knowledge of treatment. The rehabilitation will be conducted in a large busy gym where there are many different patients with different diagnoses treated at the same time by their individual physiotherapists with individual exercise programs dependent on each patient’s needs. This will reduce the probability of patients from one intervention seeing the other intervention group’s exercises. A survey on exit from the study will be conducted to determine which group the participants thought they were in during their rehabilitation.

### Procedure

Baseline and all follow-up assessments will be performed in the physiotherapy department at the rehabilitation hospital by a blinded assessor. The assessments of isometric muscle strength, knee range of movement, step tap test and 30-second chair stand test will be conducted in a closed private treatment room in the physiotherapy department. The Timed Up and Go Test and Stair Climb Test will be assessed in a corner of the gym away from the patient treatment area. The 6-minute walk and 40 m Fast-paced Walk will be assessed along a premeasured 50 m-long walkway located on the other side of the hospital. This will prevent the assessor from observing the interventions and the treating physiotherapists observing the assessments. All participants will attend daily physiotherapy for their length of stay as an inpatient, and will then attend between six and eight physiotherapy sessions as an outpatient. All participants will be given a home exercise program on discharge from inpatient rehabilitation. All participants will attend weekly outpatient physiotherapy sessions and during these sessions, the home exercise program will be progressed.

### Interventions

#### Usual care

All participants in this trial will participate in the usual care structure of rehabilitation at the treating institution. Under the usual care rehabilitation, inpatients at the hospital with TKA attend either two 45-min sessions of physiotherapy or one 45-min session of physiotherapy and one 45-min session of hydrotherapy every day on five days of the week. All participants also attend one 45-min session of either physiotherapy or hydrotherapy on the other two weekend days. All patients at the rehabilitation hospital receive hydrotherapy unless the surgeon recedes permission, or for health or personal preferences of the patient. The hydrotherapy exercises use water resistance, buoyancy and hydrotherapy equipment to achieve the same goals as the land-based therapy. Patients who do not receive hydrotherapy will attend an additional session of land-based physiotherapy, meaning that all patients receive exercise therapy twice a day. Participants will be given a home exercise program to be completed following discharge from inpatient rehabilitation. The home exercise program will be identical to the exercises performed during inpatient rehabilitation. Exercise dosage will be recorded in an exercise diary.

Participants will return to the rehabilitation hospital to participate in the outpatient rehabilitation program approximately one week following discharge from inpatient rehabilitation. All participants will attend once per week for one 45-min session of physiotherapy and either one 45-min session of hydrotherapy (if indicated) or land-based exercise (if hydrotherapy not indicated). These two 45-min sessions will occur consecutively on the same day of each week. Participants will continue their home exercise program and complete their diaries daily.

The exercises in the usual care rehabilitation program are representative of usual care following TKA, and are described in detail in Table 1 and in the Additional file 1. The goals of the usual care rehabilitation program are to improve quadriceps, hamstring and calf strength, to increase knee range of movement and to improve walking and stair climbing ability. Manual therapy including joint mobilization and massage may also be used in addition to exercises to achieve these goals.

**Table 1:** Usual Care Exercises

Goal	Exercise
Improve quadriceps strength	Static quads
	Quads over fulcrum
	Forward step-ups
	Squats
	Leg press
	Forward step-downs
Increase active knee flexion ROM	Hip and knee flexion
	Knee flexion in sitting
	Seated slides
	Exercise bike
Improve hamstring strength	Hamstring curls in prone
	Hamstring curls in standing
Increase knee extension ROM	Prone hanging
	Knee extension stretch in sitting
Improve calf muscle strength and flexibility	Heel rises
	Calf stretch

#### Intervention group

Participants in the intervention group will receive the usual care program of rehabilitation. They will also receive 15 min of additional exercises at each physiotherapy session (total time of 1 h, twice per day) designed specifically to target strengthening of the hip abductor muscles.

The following exercises are unique to the hip strengthening group and have been selected after a review of the literature investigating the efficacy of various hip abductor strengthening exercises (Table 2 and Additional file 1) [18, 19, 24–27]. They will be performed in addition to usual care received following TKA at the rehabilitation hospital. The participants will initially perform non-weight bearing antigravity hip strengthening exercises, such as sidelying hip abduction, prone hip extension, standing abduction. The exercises will be progressed to weight bearing, gravity-resisted exercises such as sideways walking, and hip abduction whilst standing on the operated leg. If the participant receives hydrotherapy they will perform the same additional hip strengthening exercises in the water, to address the identical goals of the land based program.

**Table 2:** Additional Hip Strengthening Exercises (Intervention Group)

Goal	Exercise
Improve hip strength	Sidelying hip abduction
	Prone hip extension
	Sideways walking
	Standing hip abduction
	Hip hitching

#### Control group

The participants in the control group will receive the usual care program of rehabilitation and will also receive an additional 15 min of exercise at each physiotherapy session (total session time of 1 h, twice a day) to act as a time control for the additional hip strengthening exercises prescribed for the intervention group (Table 3 and Additional file 1). These exercises have been designed to replicate functional activities such as sit-to-stand, marching and walking around a pre-measured circuit. If the participant receives hydrotherapy they will perform the same additional exercises in the water.

**Table 3:** Additional Usual Care Exercises (Control Group)

Goal	Exercise
Improve general function	Sit to stand
	Marching
	Walking

### Delivery of the intervention

Due to the physical nature of the intervention, the treating physiotherapists will not be blinded. Physiotherapists currently employed at the rehabilitation hospital will provide the interventions. The professional qualifications, years of practice and specific training prior to trial initiation will be recorded. All physiotherapists will attend a training session to ensure the exercises are performed accurately and progressed appropriately. At this training session correct and incorrect performance of the exercises will be demonstrated. Exercises will be progressed when able to be completed with minimal fatigue and no significant increase in pain. The physiotherapists will be provided with a detailed manual describing the exercise programs and progression. Follow-up training sessions will be held with the physiotherapists to ensure consistency and address any issues that may arise. Consistency and adherence will also be monitored by the recording of treatment in the participants’ medical record. The medical record includes each exercise performed, the number of repetitions of each exercise performed, any variations in the exercises performed and reasons for any variations such as adverse effects.

### Performance quality

Performance quality will be monitored by the physiotherapist. The physiotherapist will reassess each participant at the start of each session, and the exercises modified accordingly. If there are significant increases in pain (Visual Analogue Scale = 5 or greater) the physiotherapist will use his/her clinical reasoning to reduce intensity of particular aggravating exercises or add specific manual physiotherapy treatment as required. To ensure that appropriate muscle adaptation is achieved, the participant’s exercises will be progressed according to the American College of Sports Medicine (ACSM) guidelines [28] as soon as the patient is able, and details and criteria for this progression are described in the Additional file 1.

The treating physiotherapist will record the details of each treatment session, and will include the type of exercises and dosage. Any changes to the exercises or adverse responses will be noted. Following discharge from inpatient rehabilitation, participants will be given a home exercise program to continue their exercises and a diary to record daily exercises. The exercises will be progressed during the outpatient phase and the diary used to record dosage and completion of exercises. On discharge from outpatient rehabilitation, the participants will continue to complete the home exercise program until the 6-month follow-up.

### Outcome measures

Demographic information to be collected from all participants includes: name, age, gender, height, weight and body mass index. The type of prosthesis used (including patella resurfacing), left/right TKA, previous joint replacements, co-morbidities, social history (including place of accommodation, occupation/retired), co-interventions (e.g. hydrotherapy), length of hospital stay, number of treatment sessions and analgesics used will be recorded.

The measures that will be used to assess outcome are summarized in Table 4. The primary outcomes measures are: Knee Injury and Osteoarthritis Outcome score (KOOS) and isometric hip abductor muscle strength. The secondary outcome measures are: Stair Climb Test, 6 min walk test, Timed Up and Go, 40 m fast-paced walk test, 30-second chair stand test, step test, isometric quadriceps muscle strength, Lower Extremity Functional Scale (LEFS) and SF-12. These physical performance measures were selected based on the recommendations from Osteoarthritis Research Society International (OARSI) for older patients (>40 years old) diagnosed with hip and/or knee OA [29].

**Table 4:** Outcome measures

Outcome	Measurement
Knee Injury and Osteoarthritis Outcome Score	Pain, other symptoms, function and quality of life
Isometric strength of hip abductors	Hand held dynamometer (Newtons)
Stair climb test	Total time (sec) to ascend and descend 4 steps.
6 min walk test	Distance (m) walked in 6 min
Timed Up and Go	Timed rise from chair, walk 3 m and return to sitting position.
40 m fast-paced walk test	Time (sec) taken to walk 40 m
30-second chair stand test	No. of sit-to-stands performed in 30 seconds
Step test	Number of step taps on a step in 15 seconds
Isometric strength of quadriceps	Hand held dynamometer (Newtons)
Knee ROM	Goniometer (degrees)
Lower Extremity Functional Scale	Lower extremity functional status
SF-12	Overall Health
Other measures	
Compliance	Therapist treatment records and patient diary
Adverse effects	Therapist treatment records and patient diary
Type of TKR	Operation report or surgeon contact

Primary and secondary outcomes will be measured at admission to inpatient rehabilitation (approximately 7 days post-operatively), 3 weeks, 6 weeks and 6 months after commencing the rehabilitation program. A discrepancy is often reported in the recovery of patient reported outcomes compared to functional performances, with the more rapid improvement noted in patient reported outcome scales while functional performance measures are often significantly reduced in the early post-operative period [30]. Therefore, the two types of outcome measures have been included to ensure an accurate representation of rehabilitation outcomes following TKA.

### Primary outcome measures

#### Knee injury and osteoarthritis outcome score (KOOS)

The Knee Injury and Osteoarthritis Outcome Score (KOOS) is a self report questionnaire with 42-items in 5 separately analyzed subscales of pain, other symptoms, function in daily living, function in sport and recreation, and knee–related quality of life [31]. The tool has good test-retest reliability with ICC _(2,1)_ values in 26 patients 6 months post TKA of 0.73 (QOL), 0.74 (Sport/recreation), 0.82 (ADL), 0.85 (Symptoms), and 0.94 (Pain). Good content validity demonstrated by 90 % of patients who regarded improvement in the subscales Pain, Symptoms, Activities of Daily Living, and knee-related Quality of Life to be extremely or very important when deciding to have their knee operated on. The KOOS is responsive on all subscales particularly the knee-related Quality of Life subscale (effect size 2.86 at 6 months post TKA and 3.54 at 12 months post TKA) [32].

#### Isometric strength of hip abductors

The hip abductors will be tested with the participant in supine with both hips in neutral abduction and rotation. A hand held dynamometer will be used to record force, in Newtons, generated during hip abduction as per the method described previously [33]. The hand-held dynamometer (HHD) will be placed 5 cm proximal to the lateral femoral condyle. Stabilization of the HHD will be achieved by wrapping a seatbelt around the HHD and securing it to a rail on the adjacent wall at plinth height. The participants will perform a maximal voluntary isometric contraction (MVC) by abducting their limb against the dynamometer and seatbelt, held for 5 seconds. The highest of three consecutive measures will be recorded as the MVC. This method of testing hip abductor strength was reliable and valid in a sample of patients similar to those intended for the current study [33]. The seatbelt will be used to resist hip abductor strength, as this method is less susceptible to the individual strength of the therapist [34].

### Secondary outcome measures

#### Stair climb test

The stair climb test has been used extensively as an outcome measure in TKA [3, 13, 35–37]. The test assesses the time, in seconds, to ascend and descend a flight of stairs. The number of stairs in the flight and the step height are recorded. In the current study, a four-step staircase will be used. The use of a handrail and gait aids are permitted and will be recorded, as will be the footstep pattern (reciprocal or step-to). Inter-rater reliability was reported as ICC _(2,1)_ = 0.94 (95 % CI =0.55-0.98). MDC_90_ was calculated as 2.6 seconds [38]. The stair climb test has demonstrated adequate construct validity [39] and responsiveness detecting initial deterioration (effect size = −0.84) from pre-op to 1 month post-TKA scores, and then subsequent improvement (effect size = 1.26) from 1 month to 12 months post-TKA on a 12-step flight [36].

#### 6 Minute Walk Test (6 MWT)

The 6 min walk test (6MWT) is a frequently used measure for patients following TKA [3, 30, 35, 37, 40–45]. It is a test of aerobic capacity and long distance walking ability [29]. Patients are instructed to walk with usual gait aids on a premeasured circuit, covering as much distance as possible during the 6 min time frame. Rests are permitted and are included in the time. Test-retest reliability in participants with end-stage hip and knee OA awaiting joint replacement was calculated as ICC _(2,1)_ = 0.94 (95 % CI = 0.88-0.98) with standard error of measurement (SEM) of 26.3 m and MDC_90_ of 61.34 m [38]. The 6MWT is responsive when evaluating early recovery two to four months post TKA (effect size = 0.82, SRM = 1.51) [30].

#### Timed up and Go Test (TUG)

The timed up and go test (TUG) measures the time, in seconds, that a patient takes to stand from an armed chair, walk for 3 m, and return to sit on the same chair. A walking aid can be used if required [46]. The reliability of the TUG has been established in a study that combined pre-operative TKA and THA subjects. The test-retest reliability was reported as ICC _(2,1)_ =0.75 (95 % CI = 0.51-0.98) [38]. The TUG was found to be selective in its ability to distinguish healthy subjects from TKA patients [47]. It is responsive to detecting early deterioration and improvement in the early post-operative period with standardized response means (SRM) varying from −1.08 (95 % CI −1.38 to −0.92), indicating a worsening in the value to 1.04 (95 % CI 0.84 to 1.61) indicating an improvement in time taken to perform the test. Standard error of measurement (SEM) was calculated as 1.07 (95 % CI = 0.86-1.41) and minimal detectable change at the 90 % confidence level (MDC_90_) was 2.49 seconds [38].

#### 40 m Fast-paced Walk test

The purpose of the 40 m fast-paced walk test is to assess short distance walking ability [29]. The test measures the time, in seconds, taken to walk 40 m as quickly but as safely as possible excluding turns. The total time is expressed as m/s by dividing distance (40 m) by time (seconds). Any gait aids required are recorded [48]. The reliability and validity of the 40 m fast-paced walk test has been tested in a group of combined pre-operative THA and TKA patients, where test-retest reliability was found to be excellent, ICC _(2,1)_ = 0.91 (95 % CI = 0.81-0.97) and responsiveness to initial surgery followed by recovery was evident [38] Standard error of measurement (SEM) was calculated as 1.73 (95 % CI = 1.39-2.29) and minimal detectable change at the 90 % confidence level (MDC_90_) was 4.04 sec [38].

#### 30-second chair stand test

The 30-second chair stand test (30s CST) is a test of sit-to-stand ability, lower limb strength and dynamic balance [29]. Starting from a seated position, the patient stands then sits as many times as possible in 30 seconds [49]. The chair is a straight back chair with a 44 cm seat height, preferably without arms. If the patient cannot stand without using their arms, their hands can be placed on their legs and recorded as an adapted test score [29]. An initial practice trial will be performed to reduce any practice effect [50]. In a population with end stage hip or knee OA awaiting joint replacement surgery, intra-tester reliability (ICC _(1,1)=_ 0.97-0.98, 95 % CI = 0.94-0.99) and inter-tester reliability (ICC _(1,1)_ 0.93- 0.98, 95 % CI = 0.87-0.99) were excellent [50]. Convergent validity was evident by a moderate correlation (Spearman’s rho = 0.64 (95 % CI 0.49 to 0.75)) with other measures of physical function. Discriminant validity was evident by low correlation (Spearman’s rho = 0.33 (95 % CI 0.12 to 0.51)) with mental health scores. Known groups validity was demonstrated by significantly higher scores recorded for participants who ambulated without a gait aid compared to participants who did not with a mean difference of 2.8 (95 % CI 1.4 to 4.1) and effect size of 0.64 (95 % CI 0.32 to 0.95). Standardized response mean of 0.84 (95 % CI 0.61 to 1.07) indicates the 30s CST is a responsive measure [51].

#### Step test

The step test is a functional, dynamic test of standing balance [52] which has been commonly used to measure outcome of interventions in patients with OA knee [53–57]. This test is used to assess the ability of the patient to maintain standing balance whilst performing a potentially destabilizing movement [58]. The step test involves stepping one foot on, then off, a block as quickly as possible in 15 seconds without holding a support. The same procedure is then repeated with the opposite leg stepping [52]. It has high test-retest reliability in healthy older subjects (ICC > 0.90), good concurrent validity and is sensitive to changes in performance over time [52].

### Isometric strength quadriceps muscle

Isometric quadriceps strength will be measured against a strap and HHD with the patient sitting and the knee at 90° flexion [59]. Isometric quadriceps strength has been tested at knee flexion angles of 45-90° [60] and is recommended to be tested at 60-90° knee flexion [61]. Isometric quadriceps strength measured with HHD at 90° knee flexion has demonstrated excellent reliability (ICC _(2,1)_ = 0.96) and MDC_95_ of 0.58 N/kg following TKA [59]. If the participant cannot flex their knee to 90°, isometric quadriceps strength will be measured at the maximum range of flexion available and the range will be recorded. The hand-held dynamometer (HHD) will be placed on the anterior aspect of the distal tibia, 5 cm proximal to medial malleolus. Stabilization of the HHD will be achieved by wrapping a seatbelt around the HHD and securing it to a fixed attachment of the plinth. The participants will perform a maximal voluntary isometric contraction (MVC) by extending their lower leg against the dynamometer and seatbelt, held for 5 seconds. The highest of three consecutive measures will be recorded as the MVC.

### Passive knee range of movement (PROM)

Passive knee ROM will be measured with a goniometer axis placed at the lateral epicondyle of the femur, the proximal arm parallel to the long axis of the femur & pointing at the greater trochanter, the distal arm parallel to the long axis of the fibula and pointing at the lateral malleolus. Maximum passive flexion and maximum passive extension range of movement will be recorded.

### Lower Extremity Functional Scale (LEFS)

The Lower Extremity Functional Scale (LEFS) is a 20-item self-report measure of lower extremity functional status. Each item is scored on a 5-point scale (0–4) and the total LEFS scores ranges from 0 to 80 with higher scores reflecting greater levels of functional status [62]. Test-retest reliability has been reported as excellent (ICC _(2,1)_ = 0.85), SEM = 3.7 points and MDC_90_ = 9 points in a combined group TKA and THA [43, 63–66]. Internal consistency was reported as 0.93 while cross sectional validity (r = 0.68) and longitudinal validity (r = 0.64) were also good [63].

### SF-12

The SF-12 is a generic measure of a patient’s general physical and mental well-being [67] based on the SF-36 score [68]. Patients’ general and physical wellbeing can influence the outcome of joint-specific scores following TKA [69]. The SF-12 has been used extensively to measure outcomes following TKA [69–75]. Reliability coefficients of 0.84 and 0.80 were observed for the physical component and mental components respectively in a pre-operative TKA population [76]. The level of patient satisfaction with TKA patients’ pain relief and function correlated with the improvement in the physical component of the SF-12 score (r = 0.51, *P <* 0.001 and r = 0.60, *P <* 0.001) respectively. The minimal clinically important difference (MCID) for the physical component of the SF-12 was 4.5 points (95 % CI = 3.9 to 5.2) and 4.8 points (95 % CI = 4.2 to 5.4) for pain relief and function component respectively [77].

### Other measures

#### Compliance

Compliance will be assessed by review of the patient notes recorded by the treating physiotherapist. These notes will be assessed for the type of exercise, dosage of exercises and adverse events for each treatment session. On discharge from inpatient rehabilitation patients will be given an exercise diary, which will record the dosage of each exercise and any adverse events experienced daily.

#### Adverse effects

Any adverse responses will be noted in the patient notes located in the patient’s hospital file. The physiotherapists will be trained to recognize adverse events such as significant increases in knee pain, lateral hip pain in the training session held prior to the commencement of the trial.

#### Type of TKA

Details will be taken from operation report or contact with the surgeon if not recorded on operation report. The surgical approach, presence of patellar resurfacing, type of prosthesis including cruciate retaining, posterior stabilized or other, will be recorded.

#### Data and statistical analysis

An estimate of sample size was made on the ability to detect a medium effect (Cohen’s f = 0.25). The assumption of obtaining a medium effect size was reasonable based on the effect sizes reported for similar measures such as the WOMAC by other studies in the literature (effect size for WOMAC function = 0.3, WOMAC pain = 0.31) [18]. A standard statistical package was used [78]. To obtain a power of 0.8 at a significance level of α = 0.05, a minimum sample of 82 participants would be needed. To allow for loss to follow-up the aim was to recruit a sample of approximately 104 participants.

Participant characteristics and baseline data will be summarized by descriptive statistics. Independent sample t-tests for parametric data (or a chi-squared test for categorical data) will be used to determine the comparability of the two groups at baseline on measures of age, gender and BMI. All data will be analysed using intention to treat principles. Any missing data will be replaced by the last observation carried forward. Repeated measures ANOVA (group by time) calculations will be used to determine the main effects and interactions of group (hip abductor strengthening vs usual care exercises) and time (repeated factor: baseline, 3 weeks, 6 weeks, 6 months) for all outcome measures. Statistical analysis will be completed using SPSS software (version 22) and the significance level set at *P <* 0.05.

## Discussion

This study describes a protocol for a RCT that will investigate whether the addition of hip abductor muscle strengthening to the usual care rehabilitation program following TKA leads to superior performance based and patient reported outcomes. This is the first reported study to specifically assess the effect of hip strengthening exercises in this population. The trial will provide information to clinicians to potentially improve outcomes for patients with TKA. This is particularly relevant as the number of patients undergoing TKA is rapidly increasing worldwide [79–81].

Many studies striving to achieve better outcomes for TKA patients continue to focus on impairments associated directly with the knee joint. Recovery has been variable and the majority of patients continue to demonstrate lower extremity muscle weakness and functional deficits such as slower walking speeds, difficulty negotiating stairs and difficulty rising from a chair when compared to age-matched healthy individuals [13, 60, 82–84]. Therefore, it is reasonable to hypothesize that the causes of disability and poor function following TKA may also be related to other joints, particularly the hip. This hypothesis is further strengthened by the presence of hip abductor muscle weakness in patients with knee OA [14] which persists post TKA [20]. Patients with greater lower limb strength following TKA perform better on functional activities [83] demonstrating that lower limb strength contributes to functional performance. Investigations into the role of hip abductor strength following TKA have showed significant contributions of hip strength to function [21]. This is not surprising given that hip strength and hip joint mechanics have a close relationship to normal knee function. Achieving optimal outcomes following TKA therefore would require optimal hip strength in combination with optimal quadriceps strength. The investigation into targeted hip strengthening is warranted in this patient group.

It is important to evaluate the effectiveness of rehabilitation programs with appropriate outcome measures. This trial has ensured that outcome measures are included from the three different domains of physical impairment, patient reported measures, and physical performance measures, as recommended by OARSI and Mizner et al. [29, 36]. This is advantageous because many of the outcome measures for the different domains improve at different rates post-operatively. In the early stages after TKA surgery scores on the patient reported outcome scales are significantly better than preoperative scores, while functional performance outcome measures are reduced [36]. This may be due to the experience of pain free movement combined with the ability to perform a previously painful and difficult activity again resulting in high patient satisfaction and is reflected in the high scores of patient reported outcome scales [85]. The performance-based tests demonstrate an initial reduction in physical function in patients following TKA that is not seen in patient-reported measures [36]. From 1 month to 6 months postoperatively, patients’ physical performance improves [3, 43, 83] with the highest rate of improvement occurring in the period from 1 to 3 months compared with the period from 3 to 6 months [43, 35]. The outcome measures included in this trial ensure an accurate picture of recovery will be recorded.

In the evaluation of the effects of hip strengthening following TKA, it is important to determine that hip strengthening has occurred as a result of the intervention. The method of strength measurement may be considered a limitation of the study however the use of a hand-held dynamometer rather than a mechanical isokinetic dynamometer is reliable and valid with a seatbelt for restraint [33]. As this study will be conducted during the participants’ rehabilitation in a small hospital, access to an isokinetic dynamometer is not possible.

A second limitation of the study is a lack of pre-operative testing of hip abductor strength as well as other outcome measures, which would add value to the study. However, the patients that require inpatient rehabilitation in this study are not identified pre-operatively. The need for inpatient rehabilitation is identified post-operatively on an individual basis and outpatient rehabilitation is determined on arrival to the rehabilitation hospital. The lack of pre-operative testing is taken into account by measuring the hip abductor and quadriceps muscle strength at all time points in the study. The recovery of muscle strength in both groups can then be compared and their contribution to functional gains calculated.

The exercise programs described are standardized by guidelines for progression according to the American College of Sports Medicine (ACSM) guidelines [28]. Progression of exercises will also be based on the physiotherapists’ clinical assessment and tailored to the individual participants needs. The usual care exercise program has additional functional exercises to account for the extra time spent on hip strengthening exercises by the hip strengthening group participants. The use of additional manual therapy will also be decided for individual participants according to their clinical need.

There is a need for more knowledge regarding specific hip strengthening compared to usual care following TKA. The findings of this study will guide clinicians regarding the use of hip strengthening exercises in TKA rehabilitation programs.

## Abbreviations

30s CST, 30-second chair stand test; 6MWT, 6 min walk test; 95 % CI, 95 % confidence interval; ACSM, American College of sports medicine; ADL, activities of daily living; ANOVA, analysis of variance; BMI, body mass index; CONSORT, consolidated standards of reporting trials; HHD, hand held dynamometer; ICC, intra-class coefficient; KOOS, knee injury and osteoarthritis outcome score; LEFS, lower extremity functional scale; m/s, metres per second; MDC_90_, minimal detectable change at the 90 % confidence level; MVC, maximal voluntary contraction; OA, osteoarthritis; OARSI, osteoarthritis research society international; QOL, quality of life; RCT, randomized controlled trial; ROM, range of movement; SCT, stair climb test; SEM, standard error of measurement; SPIRIT, standard protocol items: recommendation for interventional trials; TKA, total knee arthroplasty; TUG, timed up and go test; WOMAC, Western Ontario and McMaster Universities arthritis index

## Additional file

Additional file 1: Exercise description and progression. **Table S1**. Usual care exercises. **Table S2**. Additional usual care exercises. **Table S3**. Additional Hip exercises. (DOCX 53194 kb)
