# Supplementary material for: Does the addition of hip strengthening exercises improve outcomes following total knee arthroplasty? A study protocol for a randomized trial
Source: BMC Musculoskelet Disord. 2016 Jun 13;17:259. doi: 10.1186/s12891-016-1104-x (PMC4906815; doi:10.1186/s12891-016-1104-x)
Supplement: Additional file 1: — Exercise description and progression. Table S1. Usual care exercises. Table S2. Additional usual care exercises. Table S3. Additional Hip exercises. (DOCX 53194 kb) [file 12891_2016_1104_MOESM1_ESM.docx]

**Additional File: Exercise description and progression**

**Table 1. Usual care exercises**

|  | **Description of exercise** | | **Starting dosage** | | | **Criteria for progression** | | **Progression** | |
| --- | --- | --- | --- | --- | --- | --- | --- | --- | --- |
|  | **Starting position** | **Movement** | **Reps and sets** | **Resistance** | **How dosage determined** | **When?** | **How known?** | **Reps and sets** | **Resistance** |
| 1. Static quads  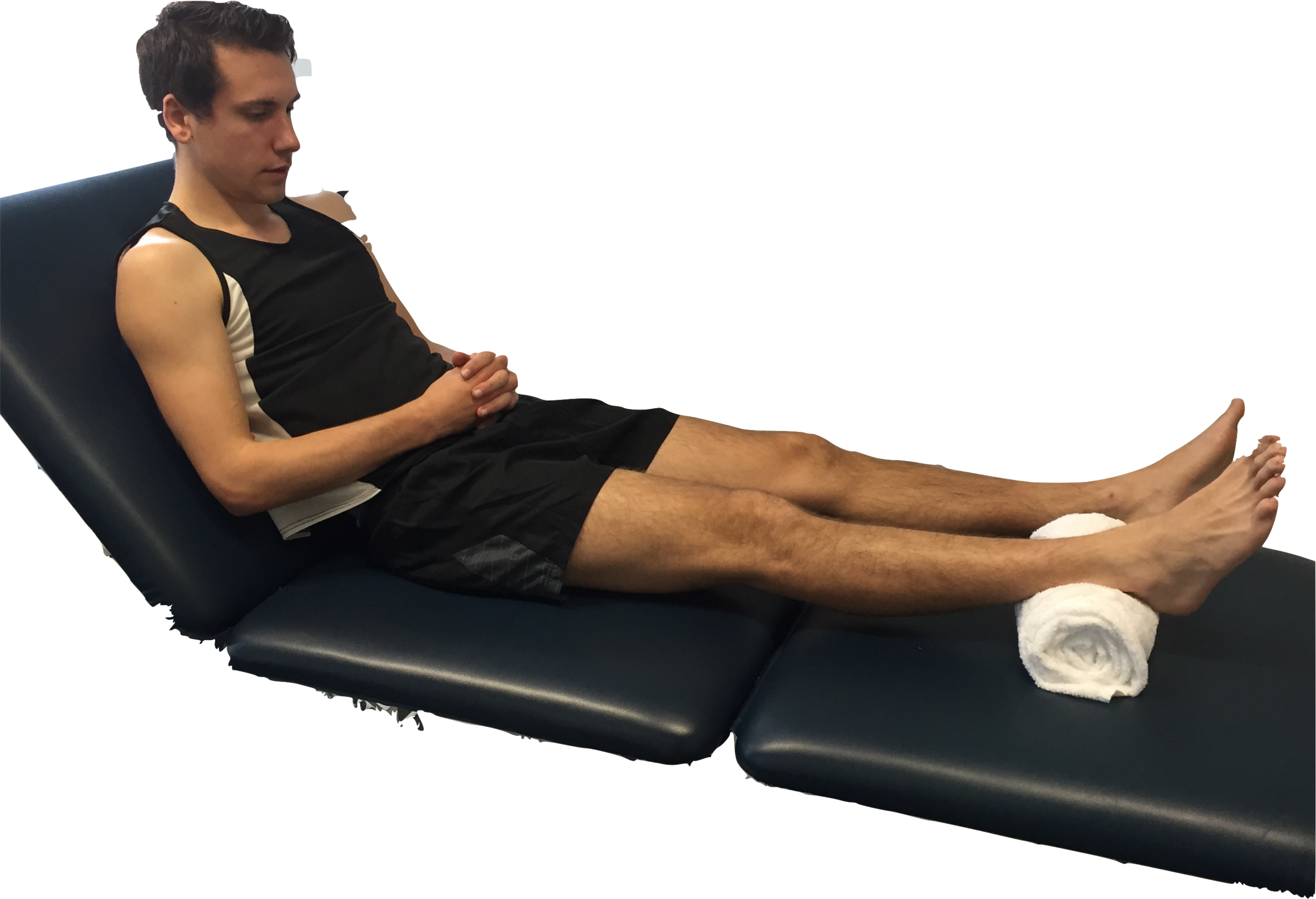 | Participant supine with rolled towel under heel | Isometric quadriceps contraction, held for 5 seconds | 1 set of 10 reps | nil | Participant should start to fatigue at rep no. 7 as measured by Borg scale of exertion level 7. | Completion with minimal fatigue, no significant increase in pain | Borg scale < 5, pain < 5/10 | 3 sets of 10 reps | Nil |
|  |  |  | 3 sets of 10 reps | nil |  | Completion with minimal fatigue, no significant increase in pain, with full range of active knee extension. | Borg scale < 5, pain < 5/10 | Cease exercise. | |
| 2. Quads over fulcrum  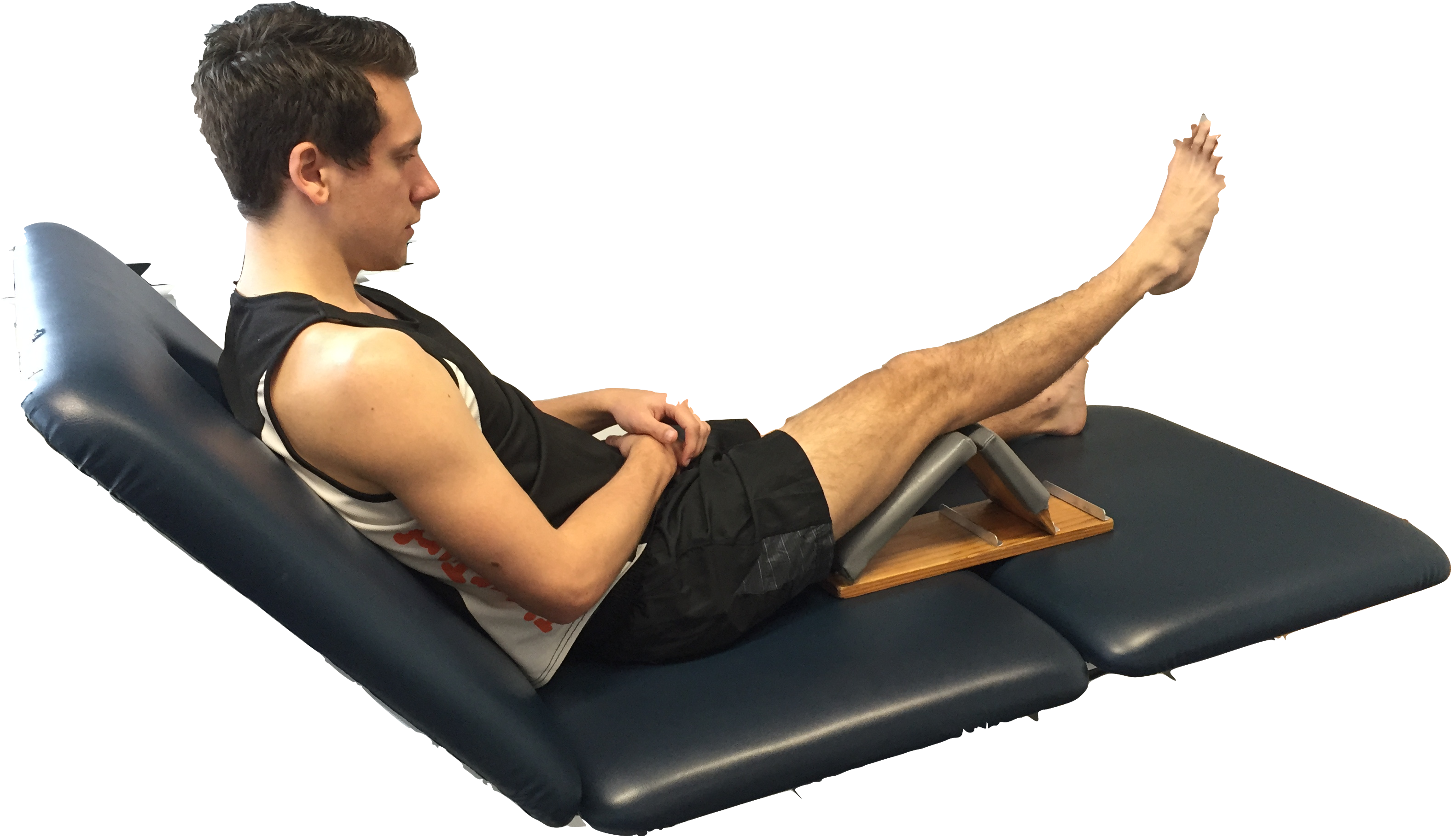 | Participant supine with knee flexed to 45° over fulcrum/roll | Knee extension to end of range | 3 sets of 10 reps | Gravity only | Participant should start to fatigue at rep no. 7 as measured by Borg scale of exertion level 7. | Completion with minimal fatigue, no significant increase in pain, with full range of active knee extension. | Borg scale < 5, pain < 5/10 | 3 sets of 10 reps | 2kg ankle weight |
|  |  |  | 3 sets of 10 reps | 2kg |  |  | Borg scale < 5, pain < 5/10 | 3 sets of 10 reps | Cease exercise. |
| 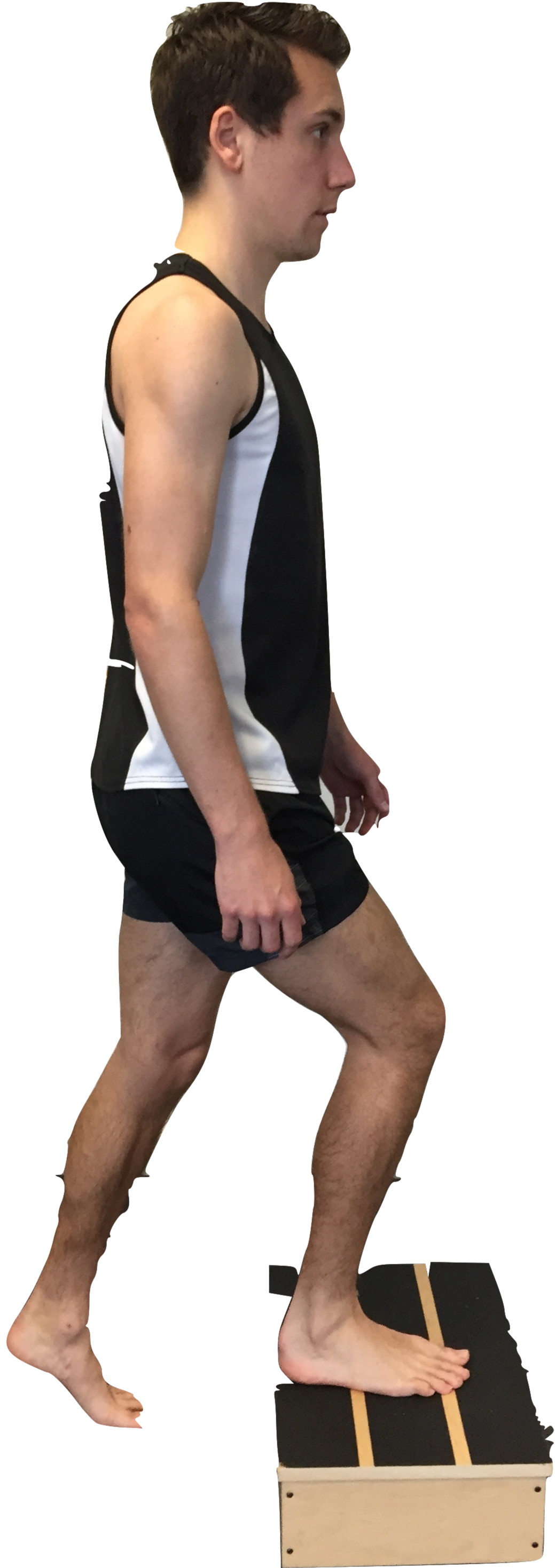3. Forward step-ups | Participant stands in front of 10cm step, holding rail for balance if necessary. | Participant steps both legs up, then down, leading with operated leg. | 1 set of 10 reps | Gravity only | Participant should start to fatigue at rep no. 7 as measured by Borg scale of exertion level 7. | Completion with minimal fatigue, no significant increase in pain | Borg scale < 5, pain < 5/10 | 3 sets of 10 reps | Gravity only |
|  |  |  | 3 sets of 10 reps | Gravity only | Participant should start to fatigue at rep no. 7 as measured by Borg scale of exertion level 7. | Completion with minimal fatigue, no significant increase in pain | Borg scale < 5, pain < 5/10 | 3 sets of 10 reps | Increase height of step to 20cm. |
| 4. Squats  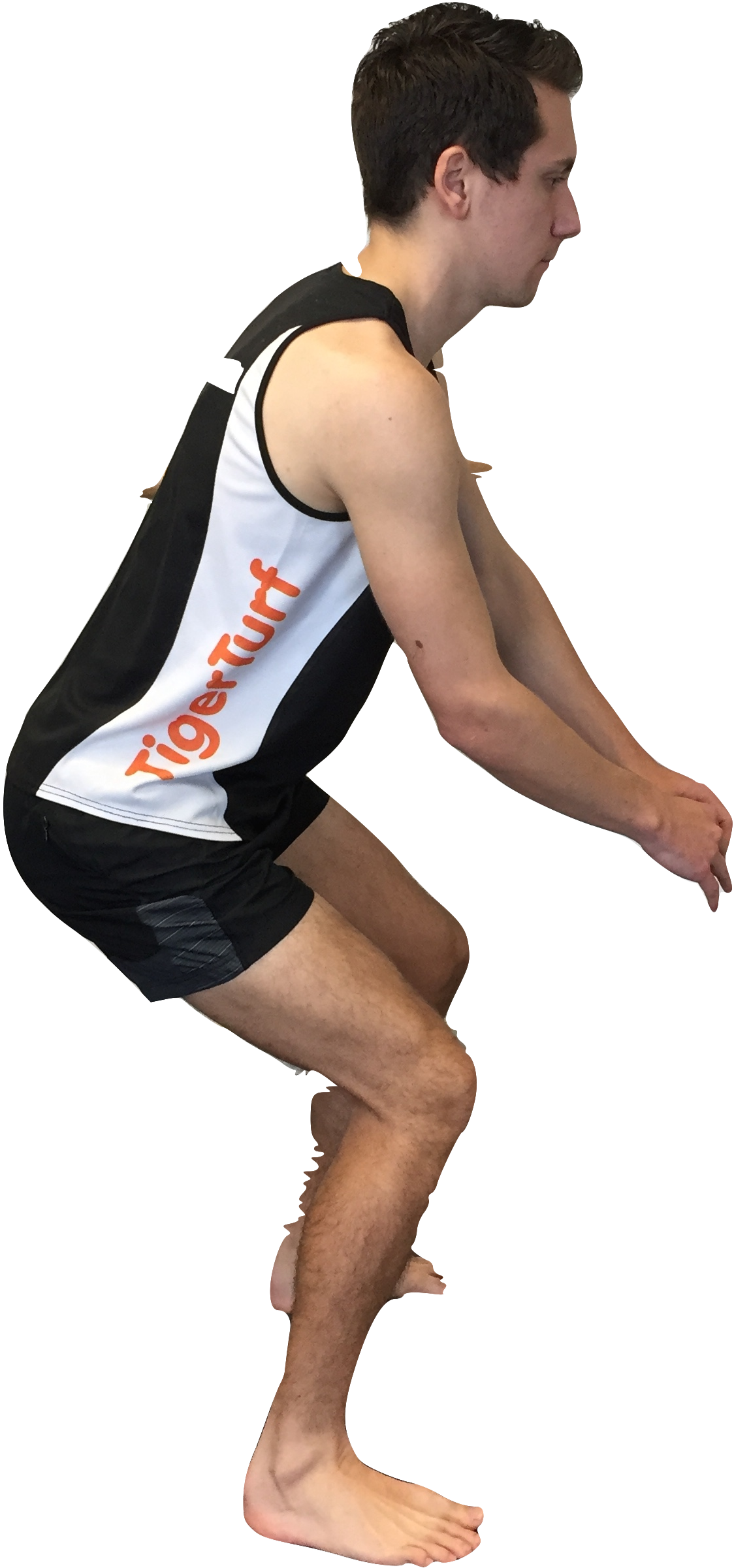 | Participant stands with feet shoulder width apart, holding rail for balance if necessary | Participant bends hips and knees as low as comfortable. Scales are used to monitor even weight distribution | 3 sets of 10 reps | Gravity only, equal weight-bearing | Participant should start to fatigue at rep no. 7 as measured by Borg scale of exertion level 7. | Completion with minimal fatigue, no significant increase in pain | Borg scale < 5, pain < 5/10 | 3 sets of 10 reps | Increase weight on operated leg to 75%, |
|  |  |  | 3 sets of 10 reps | Gravity only, 75% weight-bearing on operated leg |  | Completion with minimal fatigue, no significant increase in pain | Borg scale < 5, pain < 5/10 | 3 sets of 10 reps | Increase weight on operated leg to 90%. |
|  |  |  | 3 sets of 10 reps | Gravity only, 90% weight-bearing on operated leg |  | Completion with minimal fatigue, no significant increase in pain | Borg scale < 5, pain < 5/10 | 3 sets of 10 reps | Cease exercise. Progress to 6. Forward step downs. |
| 5. Leg press  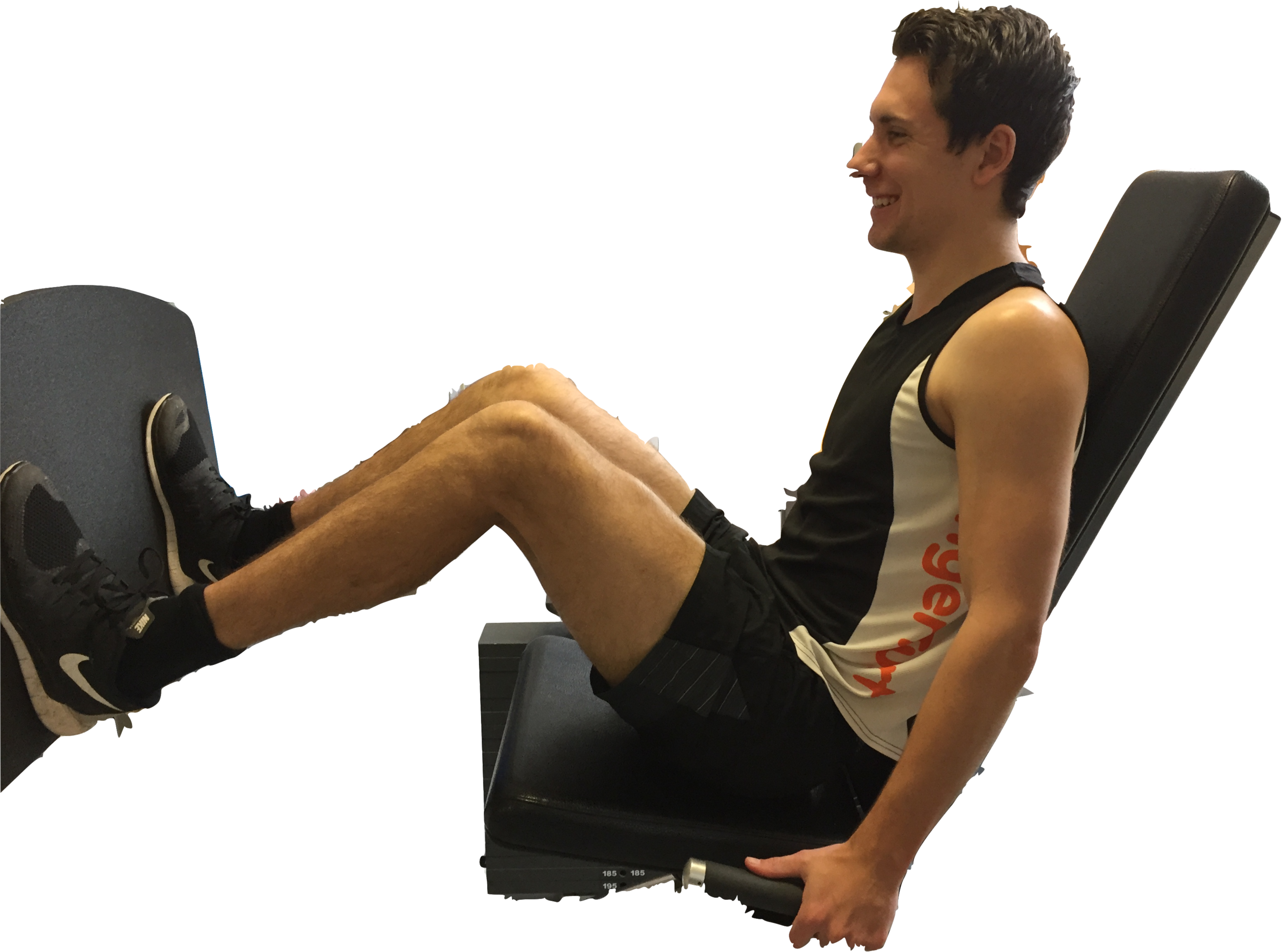 | Participant sits with both feet on the leg press platform, shoulder width apart. | Both feet are pushed against the leg press platform | 3 sets of 10 reps | 5kg | Participant should start to fatigue at rep no. 7 as measured by Borg scale of exertion level 7. | Completion with minimal fatigue, no significant increase in pain | Borg scale < 5, pain < 5/10 | 3 sets of 10 reps | Increase weight by 10% |
| 6. Forward step downs  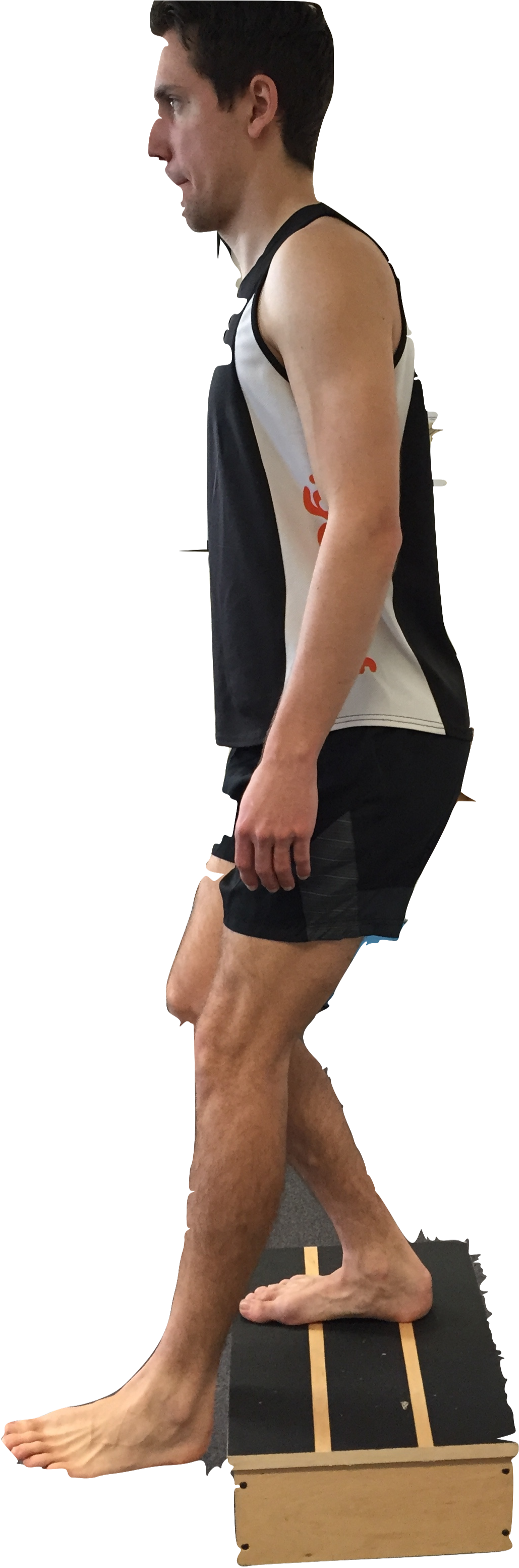 | Participant stands on 10cm step. Hold rail if necessary for balance. | Step down with non-operated leg, taps foot to ground and return. | 3 sets of 10 reps | Gravity only | Participant should start to fatigue at rep no. 7 as measured by Borg scale of exertion level 7. | Completion with minimal fatigue, no significant increase in pain | Borg scale < 5, pain < 5/10 | 3 sets of 10 reps | Increase height of step to 20cm |
| 7. Hip and knee flexion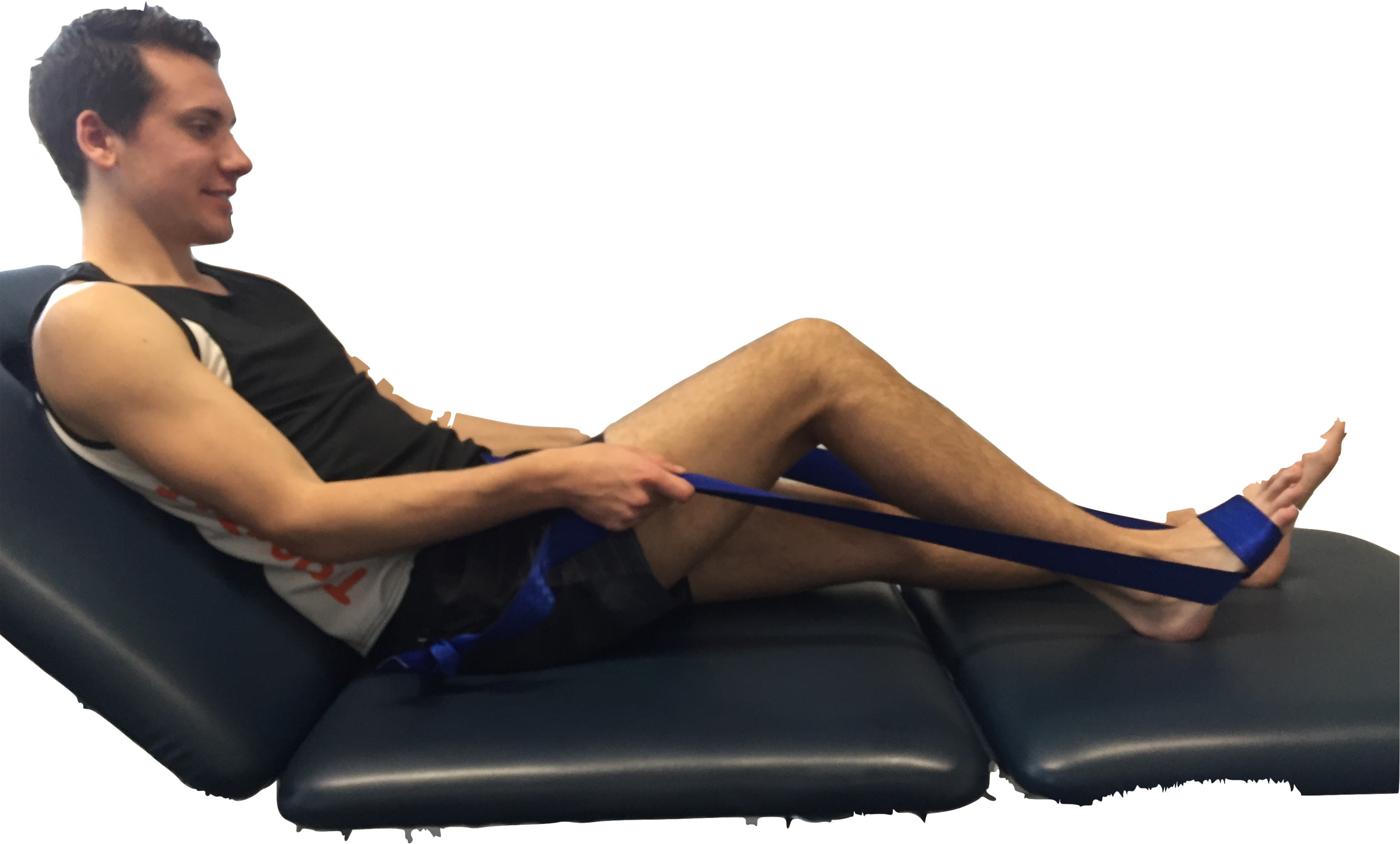 | Participant lying supine | Flexion of hip and knee, using strap around ankle to assist. | 5 second holds, 2 sets of 10 reps. | Nil | Participant should be able to move through full range of flexion and extension without significant increase in pain. (pain < 6/10) | Adequate range of knee flexion reached | Knee flexion 120° | Cease exercise | |
| 8. Knee flexion in sitting  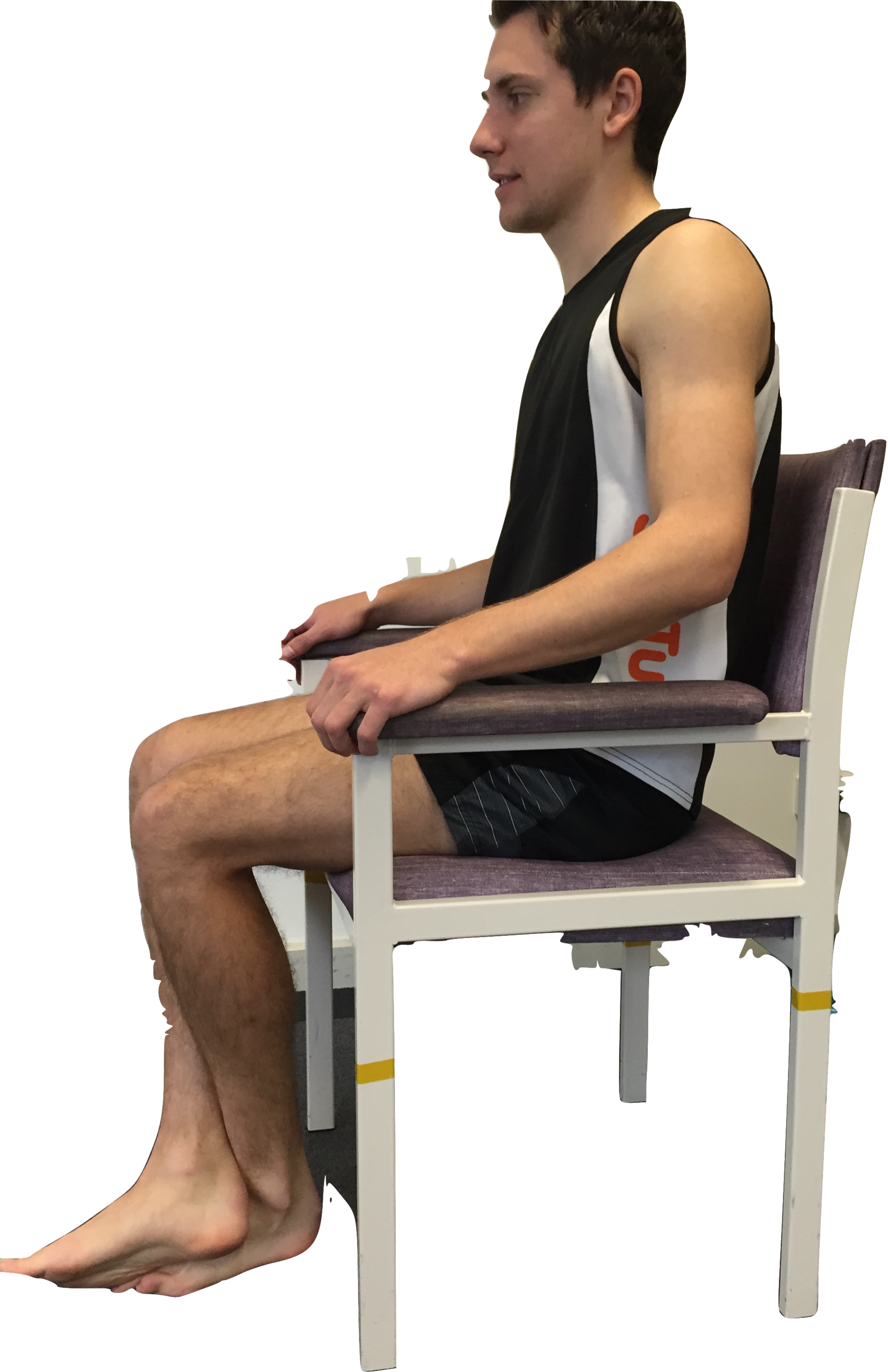 | Participant sitting in chair. | Flexion of knee | 5 second holds, 2 sets of 10 reps. | Over-pressure from other leg | Participant should be able to move through full range of flexion without significant increase in pain. (pain < 6/10) | Adequate range of knee flexion reached | Knee flexion 120° | Cease exercise | |
| 9. Seated slides  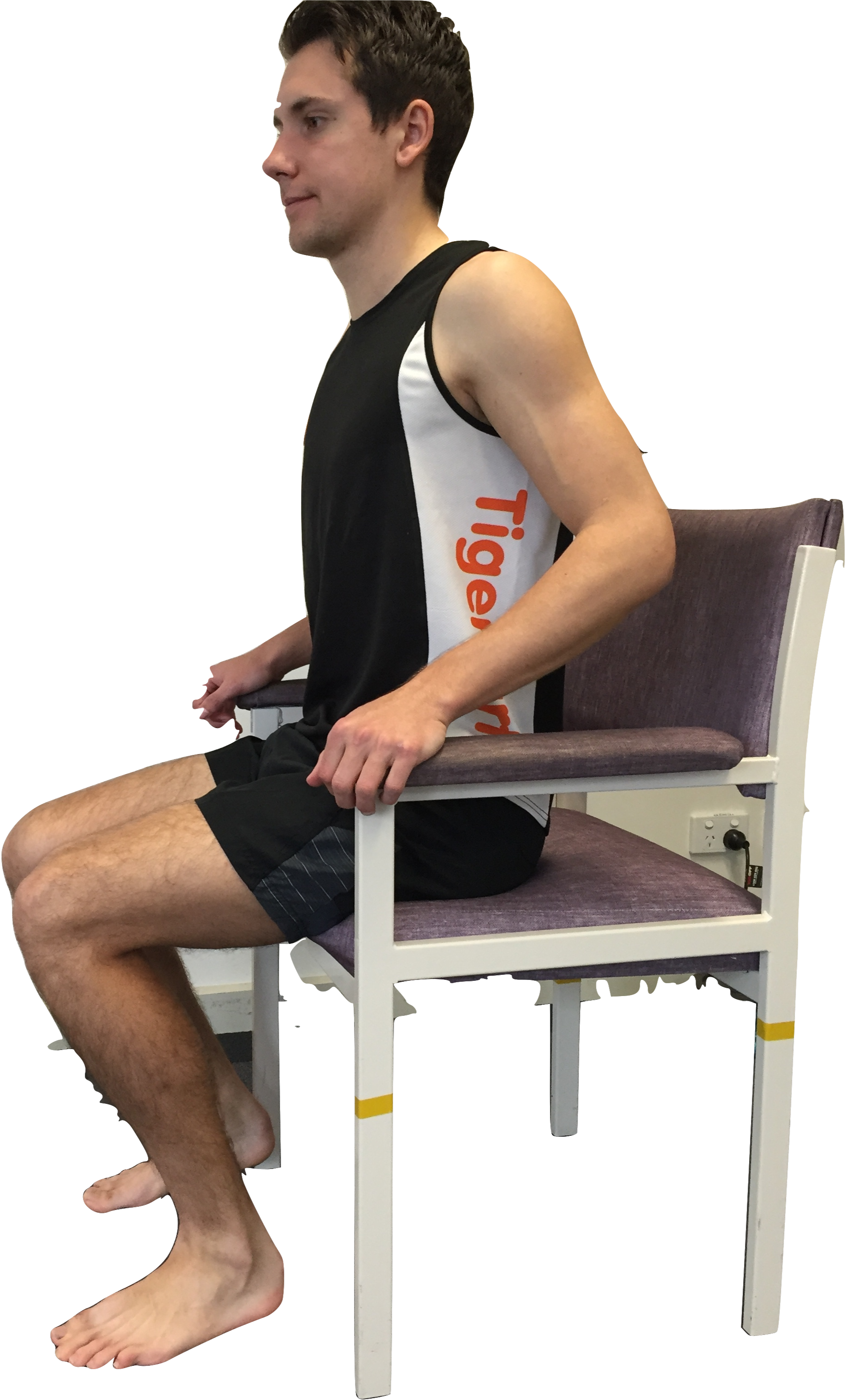 | Participant sitting in chair with knee flexed and foot on the floor. | Participant moves body forwards in the chair, keeping foot on the floor. | 5 second holds, 2 sets of 10 reps. | Nil | Participant should be able to end of available flexion range without significant increase in pain. (pain < 6/10) | Adequate range of knee flexion reached | Knee flexion 120° | Cease exercise | |
| 10. Exercise bike  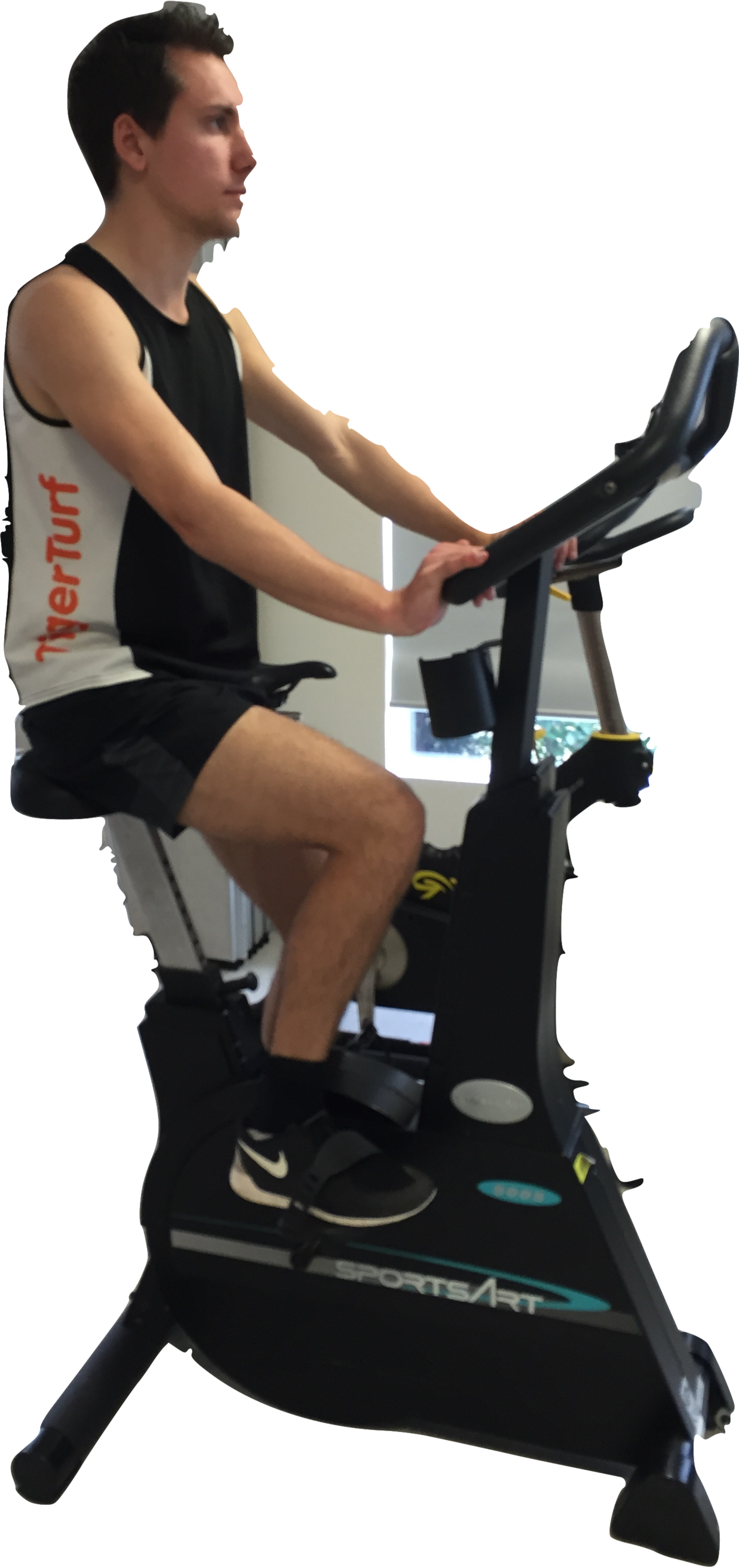 | Participant seated on bike with seat at height that allows foot to contact pedal at its lowest point. | Pedal forwards. Complete half turns if flexion range of movement inadequate for full turns. | 5 minutes, Level 1 | Nil | Participant should start to fatigue at 4 minutes as measured by Borg scale of exertion level 7. | Able to complete full turns for 5 minutes with minimal fatigue and pain | Borg scale < 5, pain < 5/10 | Increase speed to 30rpm | Level 2 |
|  |  |  | 5 minutes, Level 2 | Level 2 |  | Able to complete full turns for 5 minutes with minimal fatigue and pain | Borg scale < 5, pain < 5/10 | Lower seat height as flexion range of movement increases up to 120° | Level 2 |
| 11. Ham-string curls prone  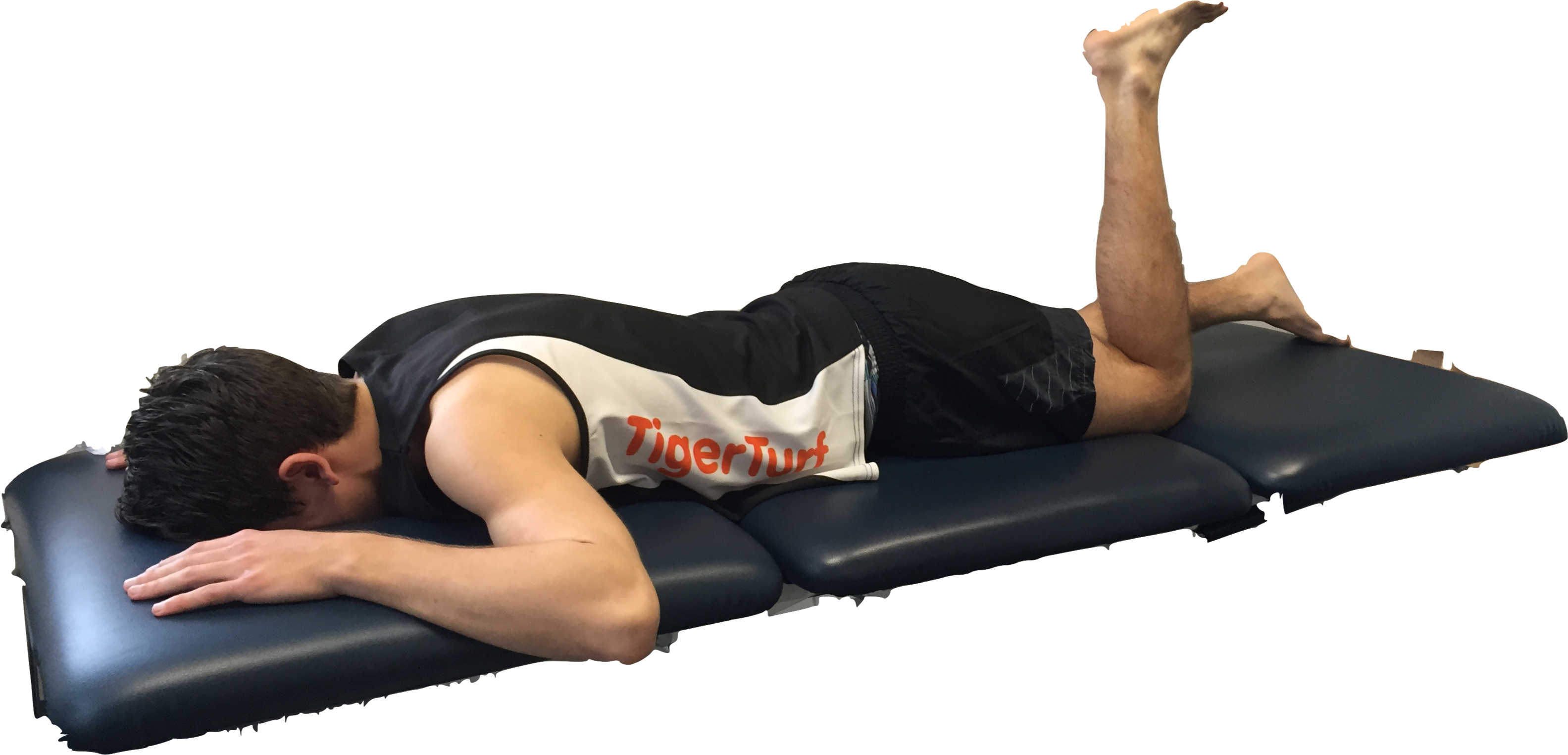 | Participant lying prone. | Knee flexion | 1 set of 10 reps | Gravity only | Participant should start to fatigue at rep no. 7 as measured by Borg scale of exertion level 7. | Completion with minimal fatigue, no significant increase in pain | Borg scale < 5, pain < 5/10 | 3 sets of 10 repetitions | Gravity only |
|  |  |  | 3 sets of 10 reps | Gravity only |  | Completion with minimal fatigue, no significant increase in pain | Borg scale < 5, pain < 5/10 | 3 sets of 10 repetitions | 2kg ankle weight. |
|  |  |  | 3 sets of 10 reps | 2kg ankle weight. |  | Completion with minimal fatigue, no significant increase in pain | Borg scale < 5, pain < 5/10 | Cease exercise | |
| 12. Ham-string curls standing  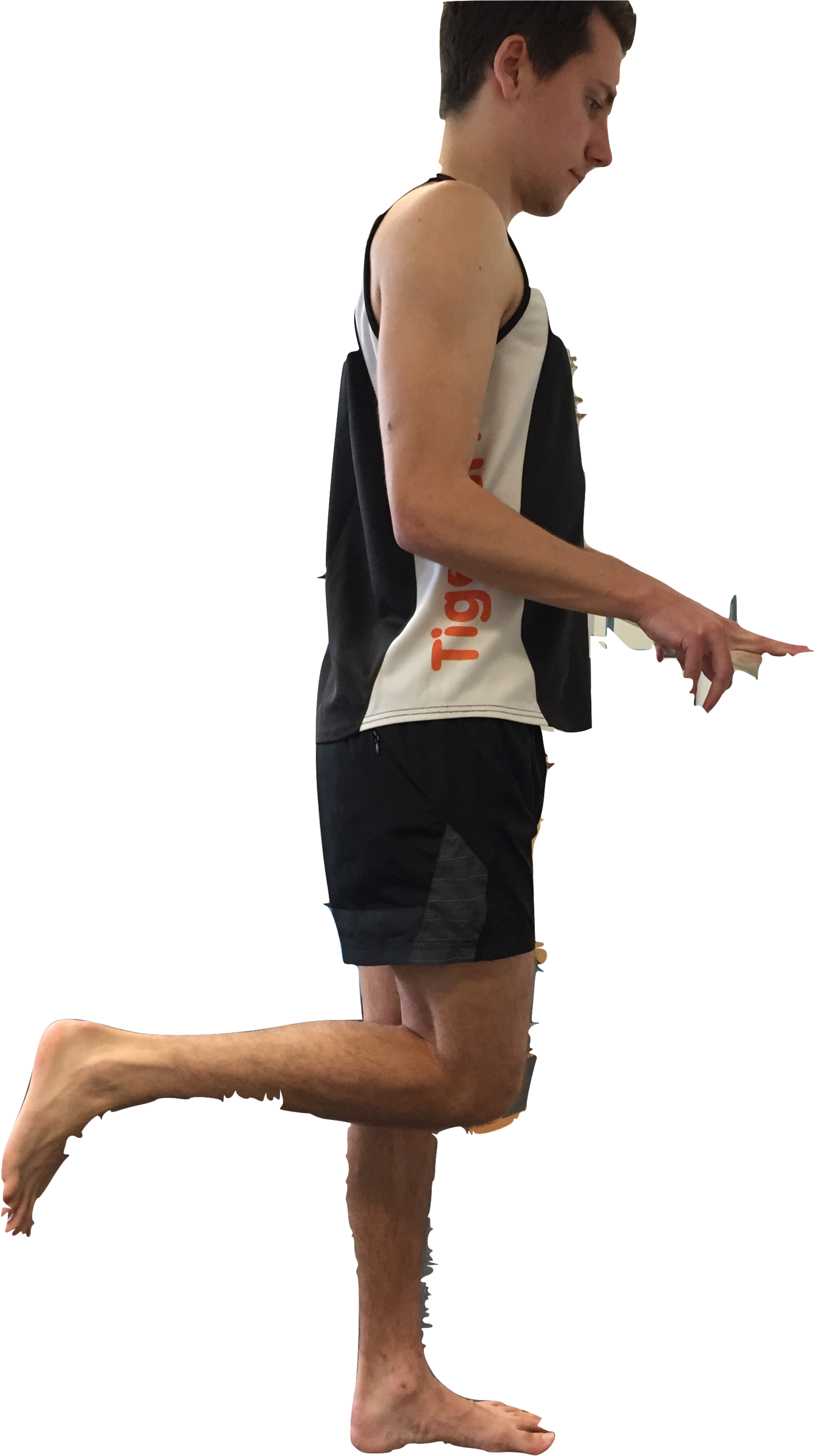 | Participant standing, holding rail for balance if required | Knee flexion | 3 sets of 10 reps | Gravity only | Participant should start to fatigue at rep no. 7 as measured by Borg scale of exertion level 7. | Completion with minimal fatigue, no significant increase in pain | Borg scale < 5, pain < 5/10 | 3 sets of 10 reps | 2kg ankle weight. |
|  |  |  | 3 sets of 10 reps | 2kg ankle weight |  | Completion with minimal fatigue, no significant increase in pain | Borg scale < 5, pain < 5/10 | Cease exercise | |
| 13. Prone hanging  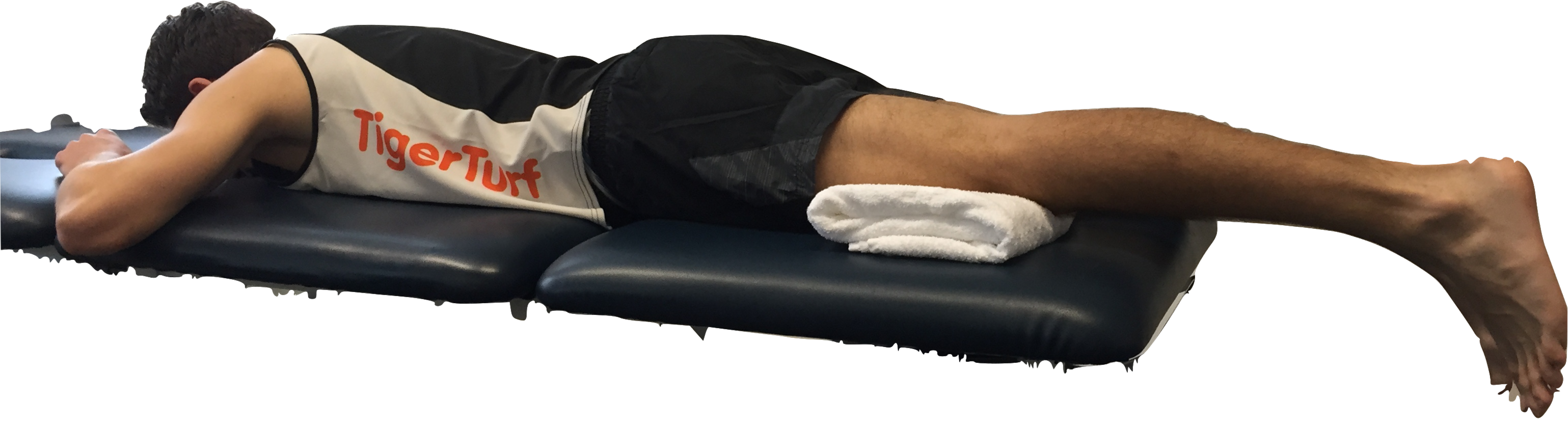 | Participant in prone with foot hanging over edge of bed. | Leg relaxed | Hold position for 60 seconds | Gravity only | Participant should feel stretch at posterior aspect of knee. | Full knee extension not achieved | Knee extension <0° | Increase time of hanging by 1minute each time up to 5 minutes. | Gravity only |
|  |  |  | Hold position for 5 minutes | Gravity only |  | Full knee extension not achieved | Knee extension <0° | 5 minutes | 2 kg ankle weight |
| 14. Knee extension stretch sitting  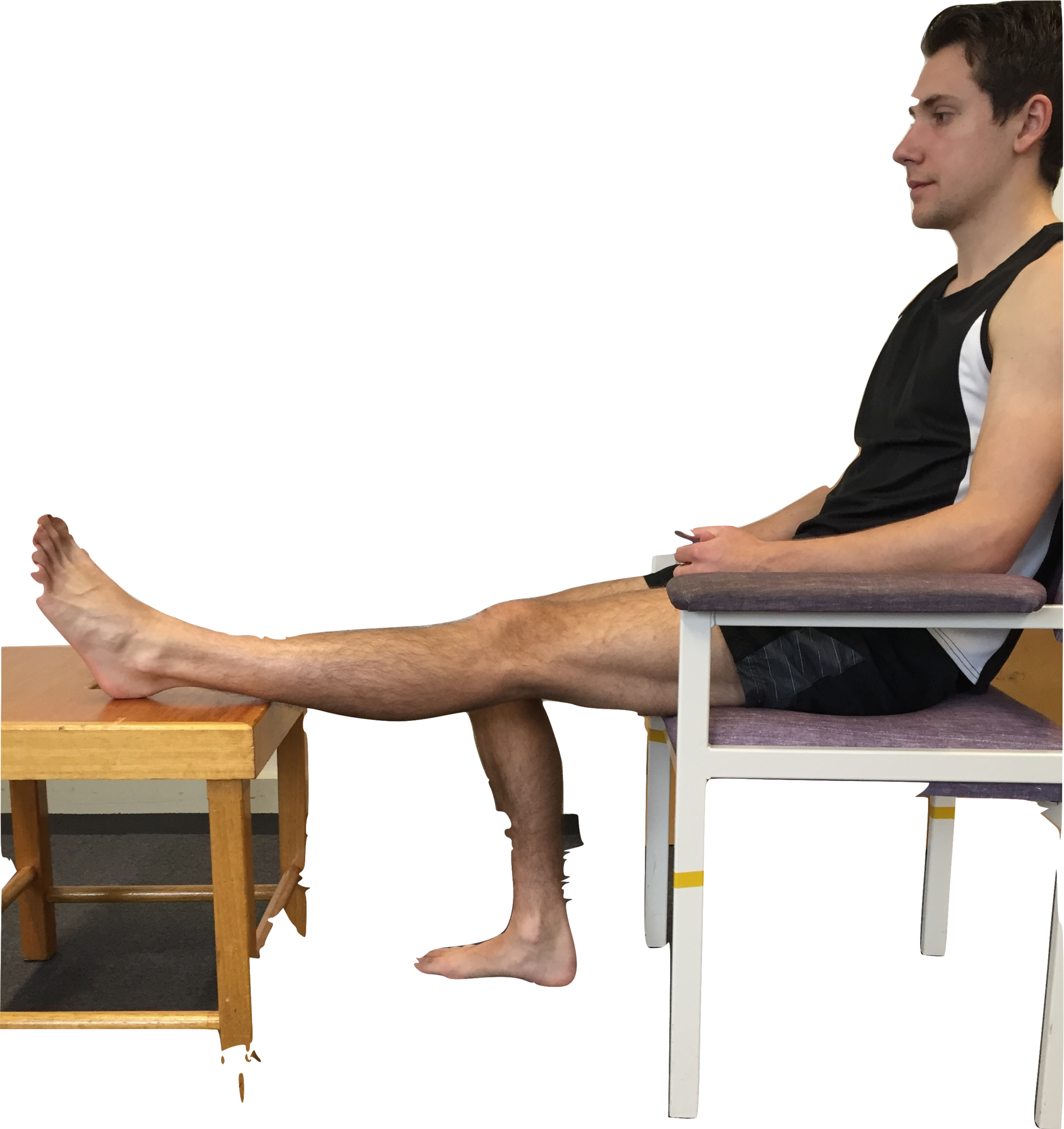 | Participant in sitting with foot resting out straight on another chair | Leg relaxed | Hold position for 60 seconds | Gravity only | Participant should feel stretch at posterior aspect of knee. | Full knee extension not achieved | Knee extension <0° | Increase time by 1minute each time up to 5 minutes. | Gravity only |
|  |  |  | Hold position for 5 minutes | Gravity only |  | Full knee extension not achieved | Knee extension <0° | 5 minutes | 2 kg weight across the knee |
| 15. Heel raises  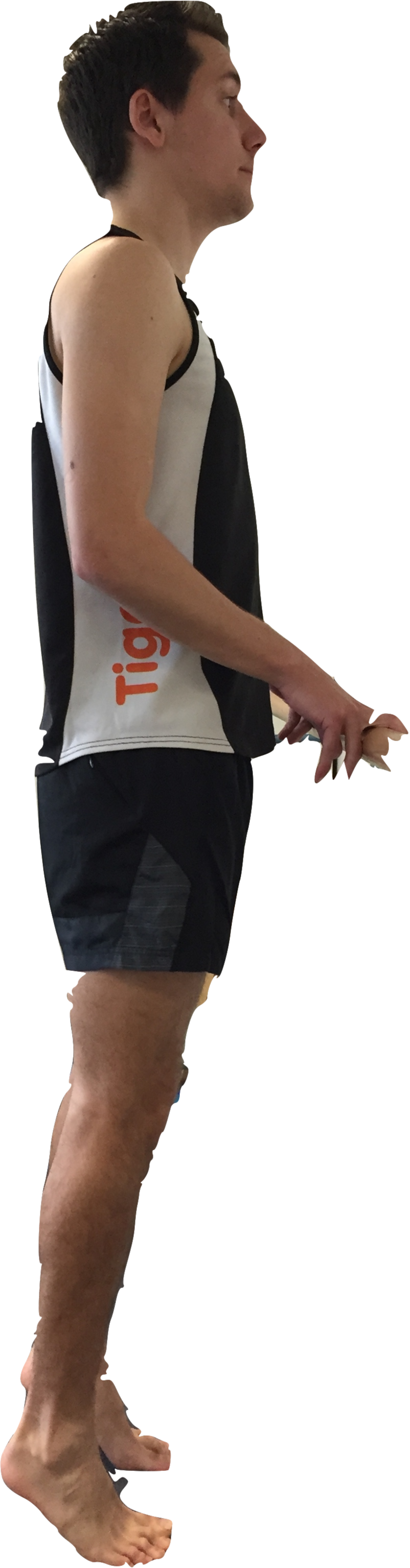 | Participant in standing holding rail for balance if necessary | Rise up onto toes. Scales are used to determine equal weight bearing. | 3 sets of 10 reps | Gravity only, 50% weightbearing on operated leg | Participant should start to fatigue at rep no. 7 as measured by Borg scale of exertion level 7. | Completion with minimal fatigue, no significant increase in pain | Borg scale < 5, pain < 5/10 | 3 sets of 10 reps | Increase weight on operated leg to 75% |
|  |  |  | 3 sets of 10 reps | Gravity only, 75% weightbearing on operated leg |  | Completion with minimal fatigue, no significant increase in pain | Borg scale < 5, pain < 5/10 | 3 sets of 10 reps | Increase weight on operated leg to 90%, then 100% |
| 16. Calf stretch  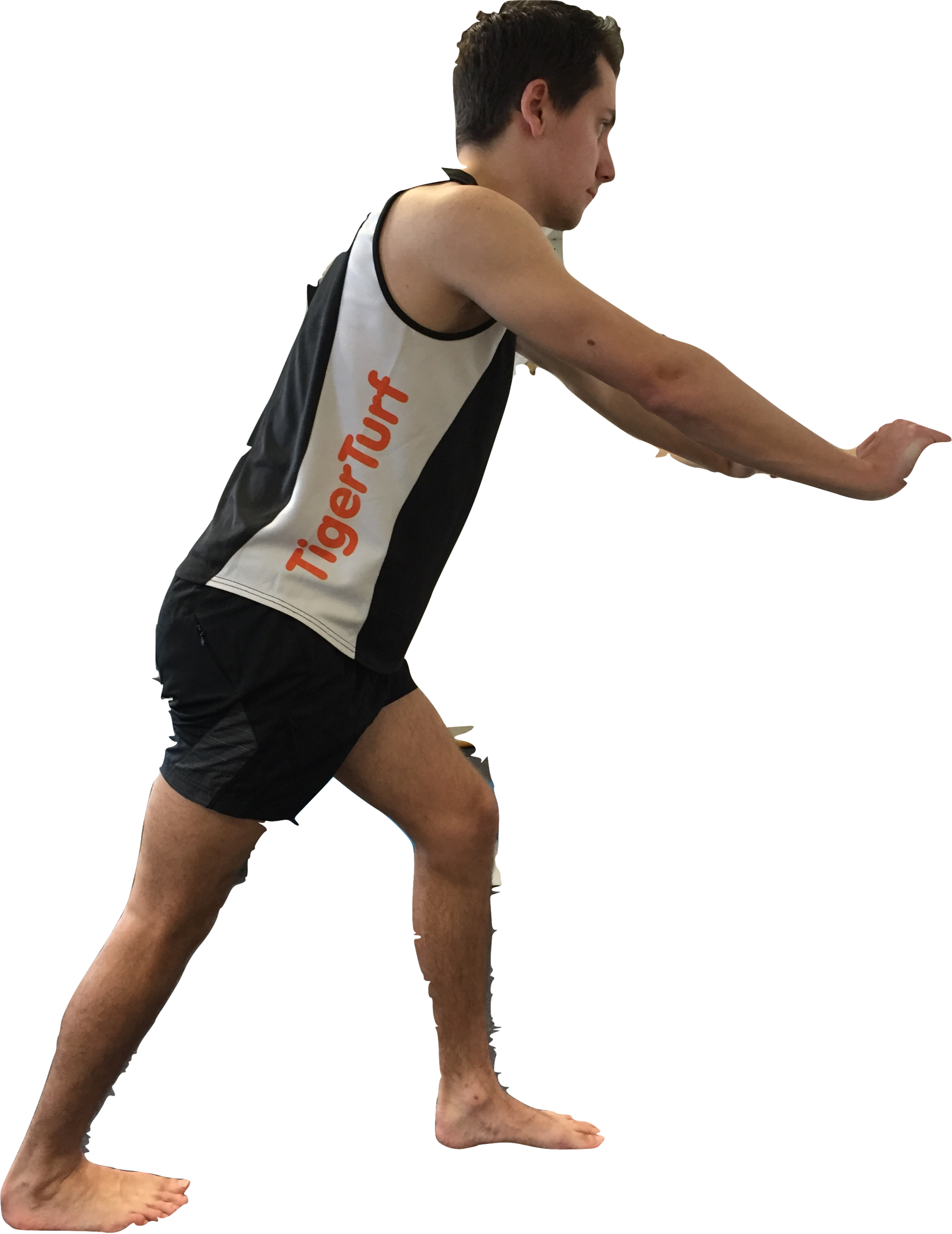 | Participant in standing with the operated leg straight out behind, foot flat and toes facing forwards. | Flexion of non-operated knee to lean forwards | Hold 30 seconds, 5 reps | Nil |  | N/a | | | |

Reps, repetitions; rpm, revolutions per minute; Borg Scale, (0-10) scale.

**Table 2. Additional usual care exercises**

| **Exercise** | **Description of exercise** | | **Starting dosage** | | | **Criteria for progression** | | **Progression** | |
| --- | --- | --- | --- | --- | --- | --- | --- | --- | --- |
|  | **Starting position** | **Movement** | **Reps and sets** | **Resistance** | **How dosage determined** | **When?** | **How known?** | **Reps and sets** | **Resistance** |
| 1. Sit to stand  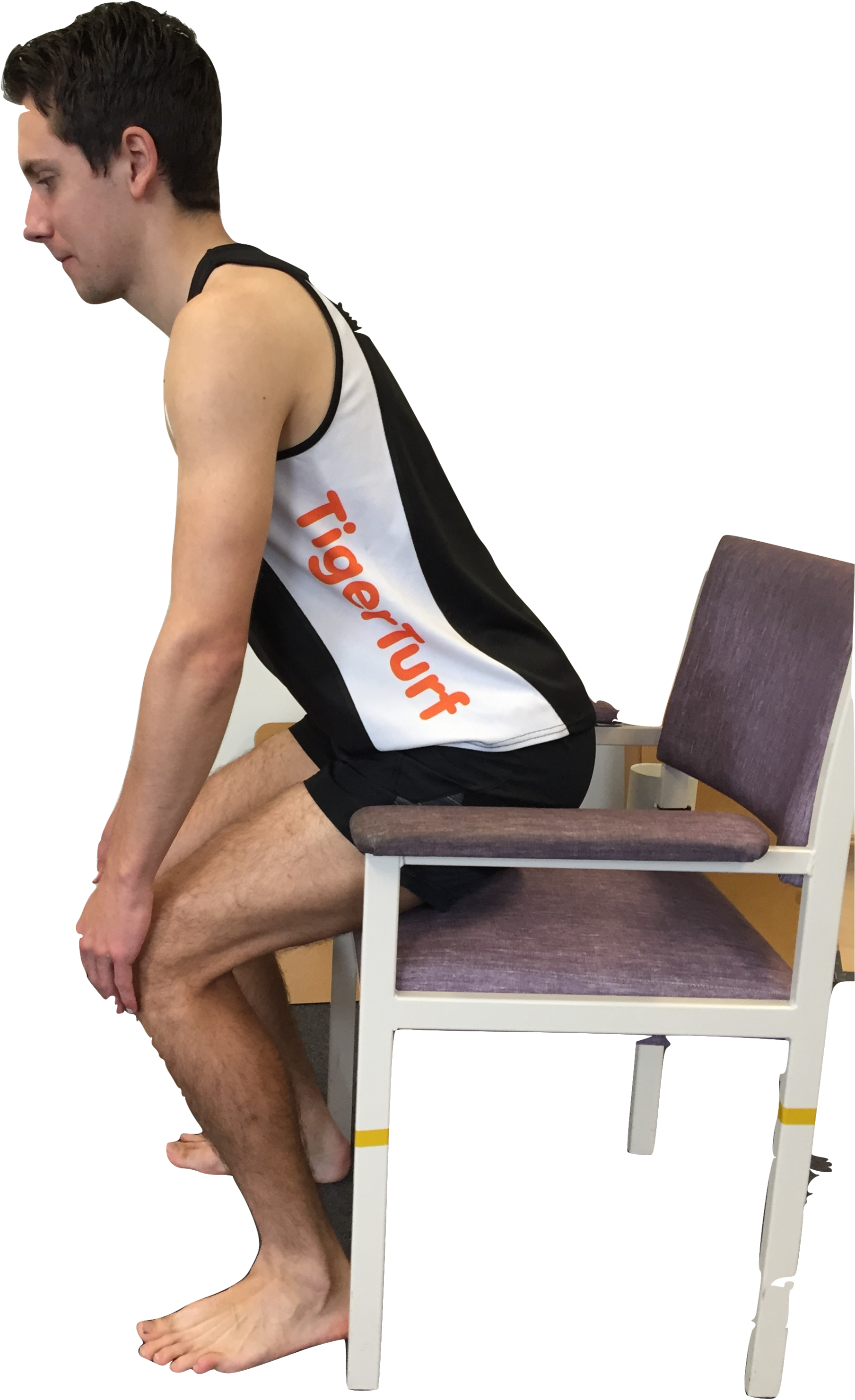 | Participant in sitting | Stand up then sit down. Use arms if necessary | 1 sets of 10 reps | Gravity only | Participant should start to fatigue at rep no. 7 as measured by Borg scale of exertion level 7. | Completion with minimal fatigue, with no significant increase in pain | Borg scale < 5, pain < 5/10 | 3 sets of 10 reps | Gravity only |
|  |  |  | 3 sets of 10 reps | Gravity only |  |  |  | 3 sets of 10 reps | Gravity only, no assistance from arms. |
| 2. Marching  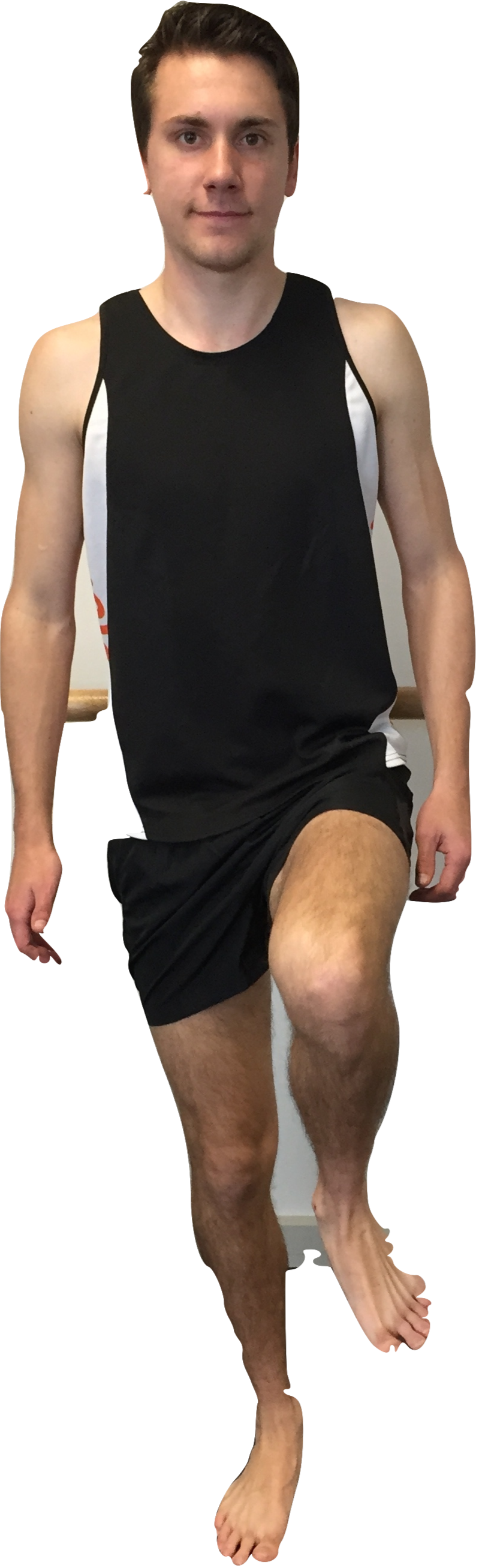 | Participant standing, near rail for balance if necessary | Participant marches on the spot | I minute | Nil | Participant should start to fatigue at rep no. 7 as measured by Borg scale of exertion level 7. | Completion with minimal fatigue, with no significant increase in pain | Borg scale < 5, pain < 5/10 | Increase by 1 minute increments up to 5 minutes | Nil |
| 3. Walking | Participant in standing with gait aids as required | Participant walks around 120m circuit | 1 lap of circuit | Nil | Participant should start to fatigue at rep no. 7 as measured by Borg scale of exertion level 7. | Completion with minimal fatigue, with no significant increase in pain | Borg scale < 5, pain < 5/10 | Increase by 1 lap each progression | Nil |

Reps, repetitions; rpm, revolutions per minute; Borg Scale, (0-10 scale).

**Table 3. Additional Hip exercises**

| **Exercise** | **Description of exercise** | | **Starting dosage** | | | **Criteria for progression** | | **Progression** | |
| --- | --- | --- | --- | --- | --- | --- | --- | --- | --- |
|  | **Starting position** | **Movement** | **Reps and sets** | **Resistance** | **How dosage determined** | **When?** | **How known?** | **Reps and sets** | **Resistance** |
| 1. Side lying hip abduction  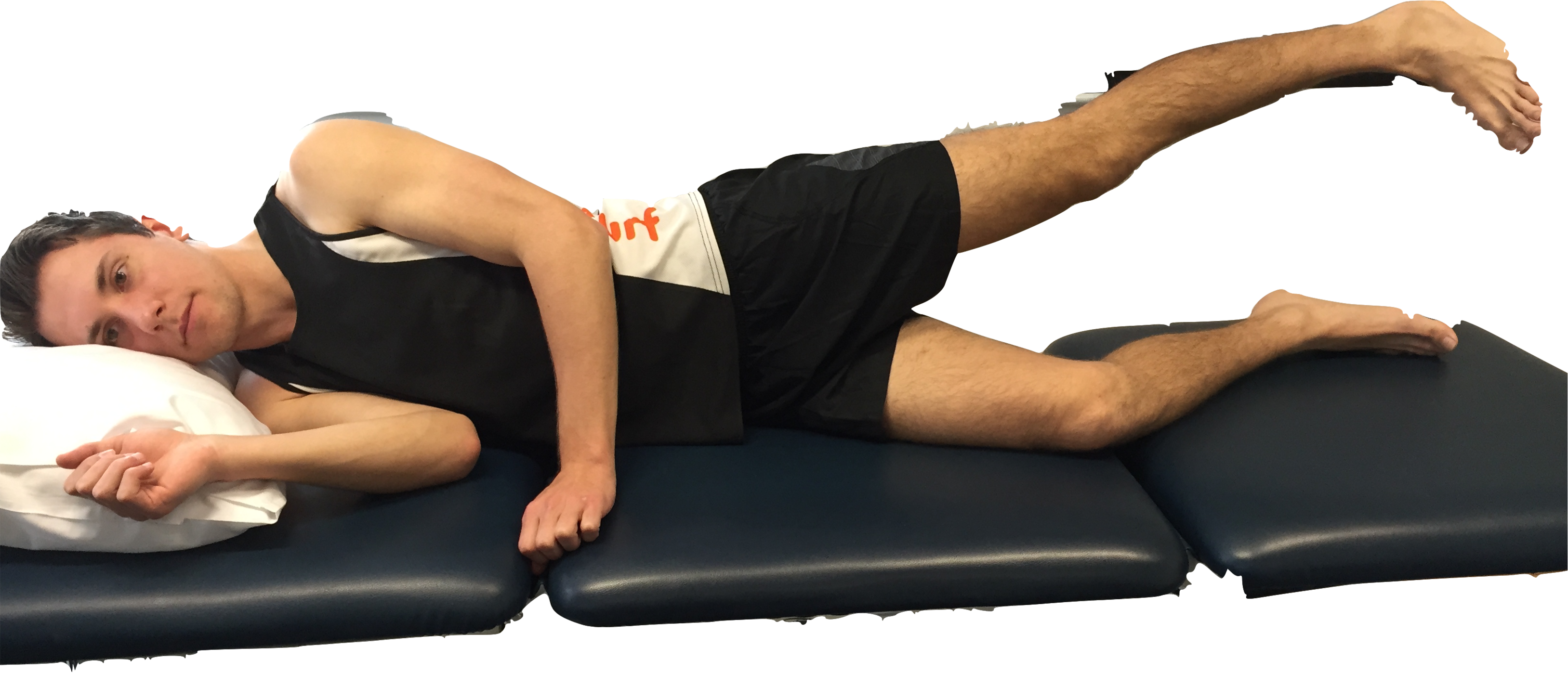 | Participant in sidelying with operated limb uppermost | Abduction of hip to 30° and return. Avoid external hip rotation or flexion. | 1 set of 10 reps | Gravity only | Participant should start to fatigue at rep no. 7 as measured by Borg scale of exertion level 7. | Completion with minimal fatigue, no significant increase in pain | Borg scale < 5, pain < 5/10 | 3 sets of 10 reps | Gravity only |
| 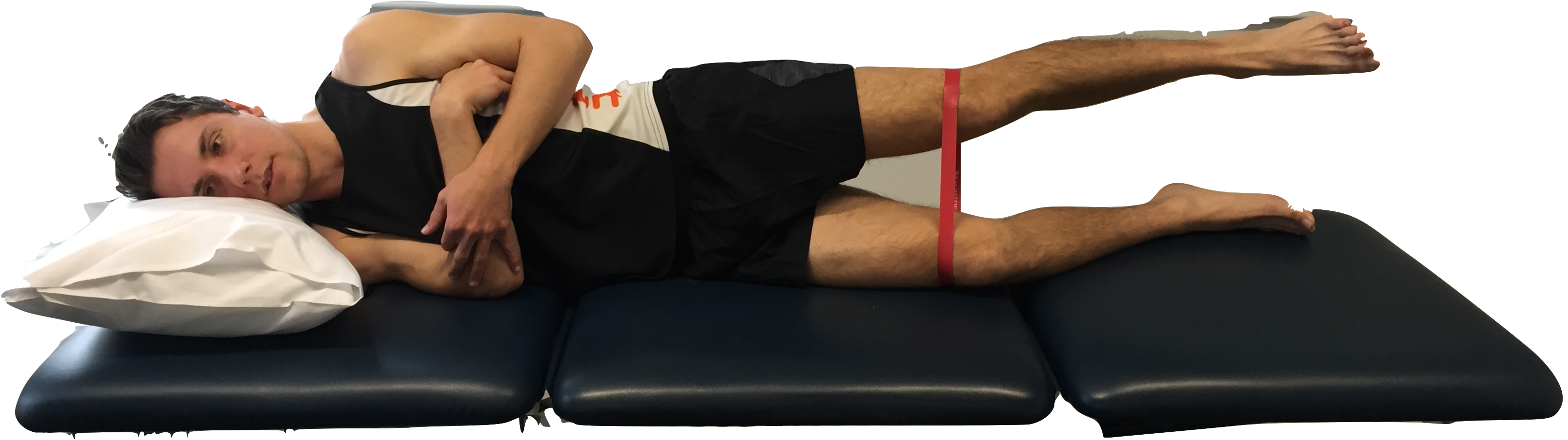 |  |  | 3 sets of 10 reps | Gravity only |  | Completion with minimal fatigue, no significant increase in pain | Borg scale < 5, pain < 5/10, no hip pain | 3 sets of 10 reps | Red theraband |
|  |  |  | 3 sets of 10 reps | Red theraband |  | Completion with minimal fatigue, no significant increase in pain | Borg scale < 5, pain < 5/10, no hip pain | 3 sets of 10 reps | Replace red theraband with blue theraband. Continue progression through theraband resistance applying similar progression criteria. |
|  |  |  | 3 sets of 10 reps | Black theraband |  | Completion with minimal fatigue, no significant increase in pain | Borg scale < 5, pain < 5/10, no hip pain | Cease exercise | |
| 2. Prone hip extension  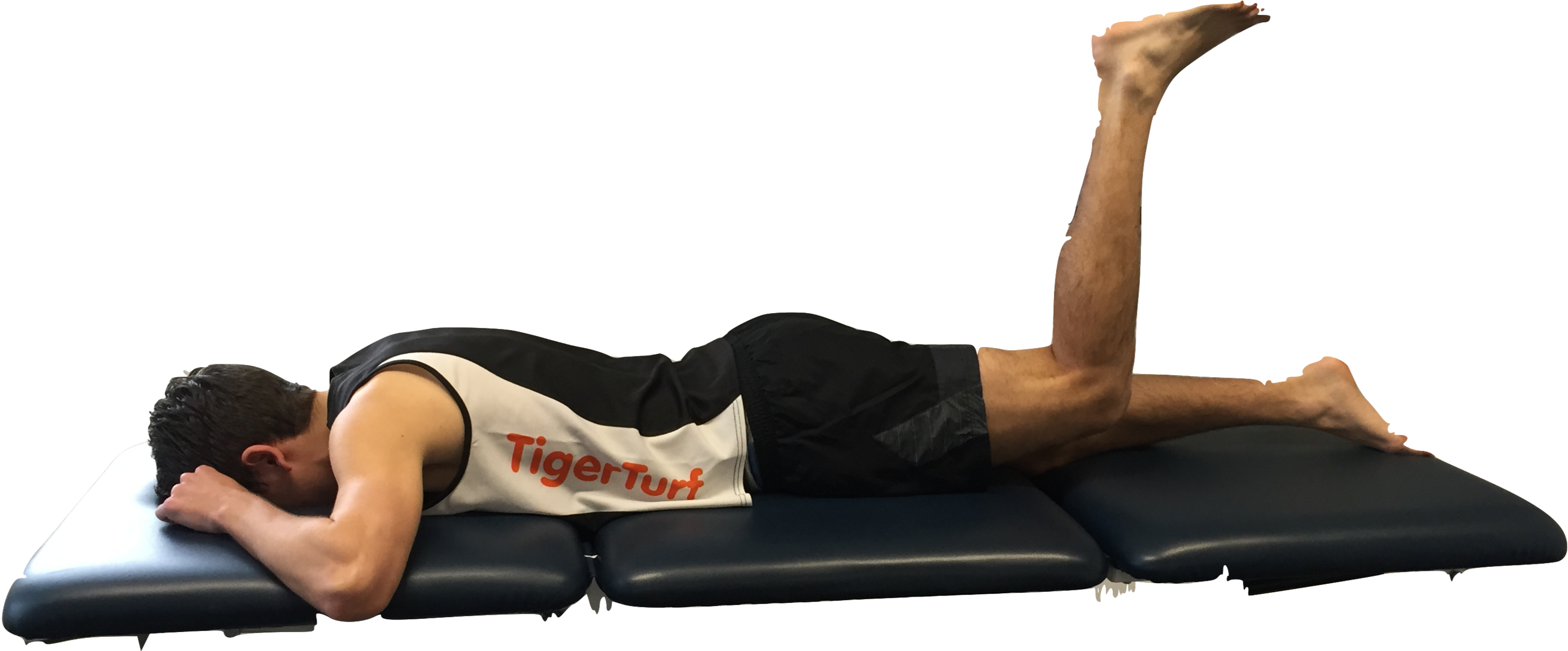 | Particpant in prone with knee flexed as close to 90° as possible | Extension of hip to 10° extension and return | 1 set of 10 reps | Gravity only | Participant should start to fatigue at rep no. 7 as measured by Borg scale of exertion level | Completion with minimal fatigue, no significant increase in pain | Borg scale < 5, pain < 5/10 | 3 sets of 10 reps | Gravity only |
|  |  |  | 3 sets of 10 reps | Gravity only |  | Completion with minimal fatigue, no significant increase in pain | Borg scale < 5, pain < 5/10 | 3 sets of 10 reps | 2 kg ankle weight |
|  |  |  | 3 sets of 10 reps | 2 kg ankle weight |  | Completion with minimal fatigue, no significant increase in pain | Borg scale < 5, pain < 5/10 | Cease exercise | |
| 3. Sideways walking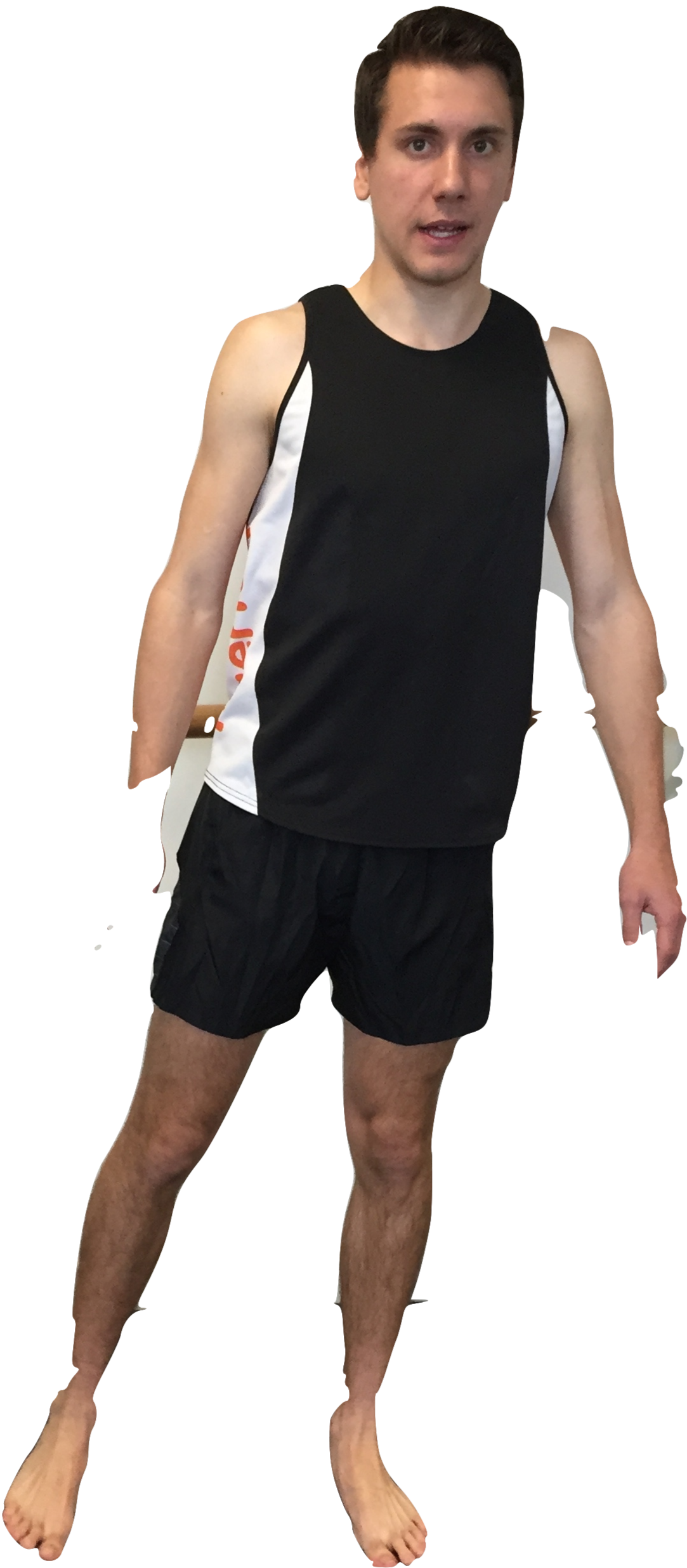 | Participant in standing, holding rail for balance if necessary. | Walk sideways 5m, avoiding hip external rotation. | Repeat 10 times. | Nil | Participant should start to fatigue at rep no. 7 as measured by Borg scale of exertion level 7. | Completion with minimal fatigue, no significant increase in pain | Borg scale < 5, pain < 5/10 | Repeat 10 times. | Red theraband |
| 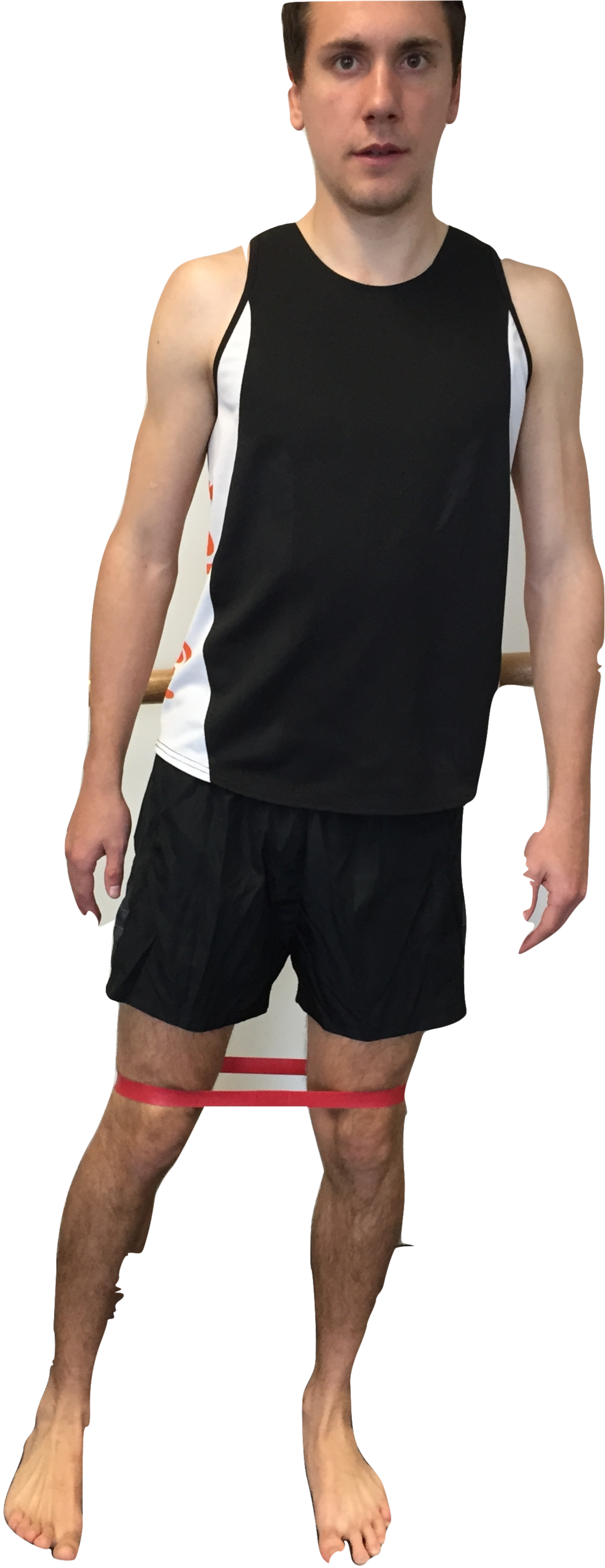 |  |  |  |  |  | Completion with minimal fatigue, no significant increase in pain | Borg scale < 5, pain < 5/10 | Repeat 10 times. | Replace red theraband with blue theraband. Continue progression through theraband resistance applying similar progression criteria. |
| 4. Standing hip abduction  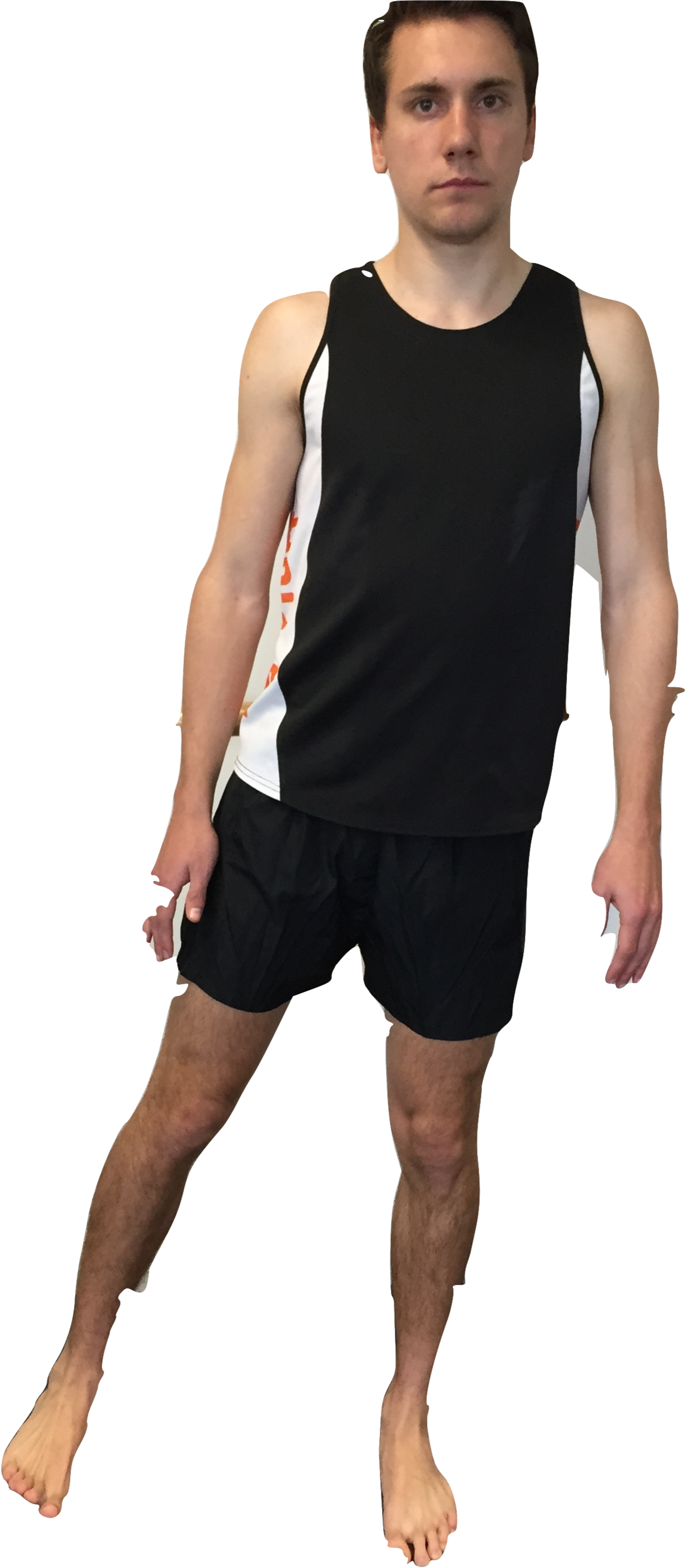 | Participant standing on non-operated leg, holding rail for balance if necessary. | Abduction of hip to 30° and return. Avoid external hip rotation or flexion. | 3 sets of 10 reps | Nil | Participant should start to fatigue at rep no. 7 as measured by Borg scale of exertion level 7. | Completion with minimal fatigue, able to stand on operated leg with no significant increase in pain | Borg scale < 5, pain < 5/10 | 3 sets of 10 reps | Repeat standing on operated leg. |
| 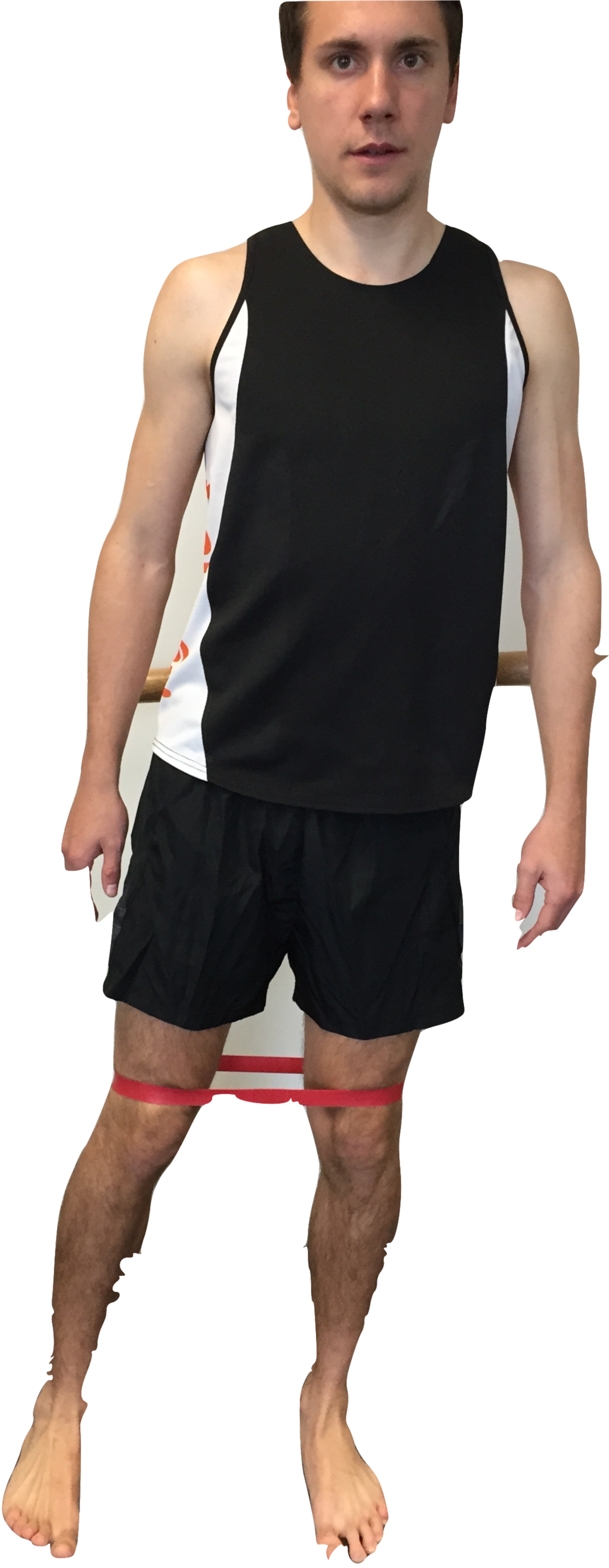 | Participant standing on operated leg, holding rail for balance if necessary. | Abduction of hip to 30° and return. Avoid external hip rotation or flexion. Repeat on other leg | 3 sets of 10 reps | Nil |  | Completion with minimal fatigue, with no significant increase in pain | Borg scale < 5, pain < 5/10 | 3 sets of 10 reps | Red theraband |
|  |  |  | 3 sets of 10 reps | Red theraband |  | Completion with minimal fatigue, with no significant increase in pain | Borg scale < 5, pain < 5/10 | 3 sets of 10 reps | Replace red theraband with blue theraband. Continue progression through theraband resistance applying similar progression criteria. |
| 5. Hip hitching  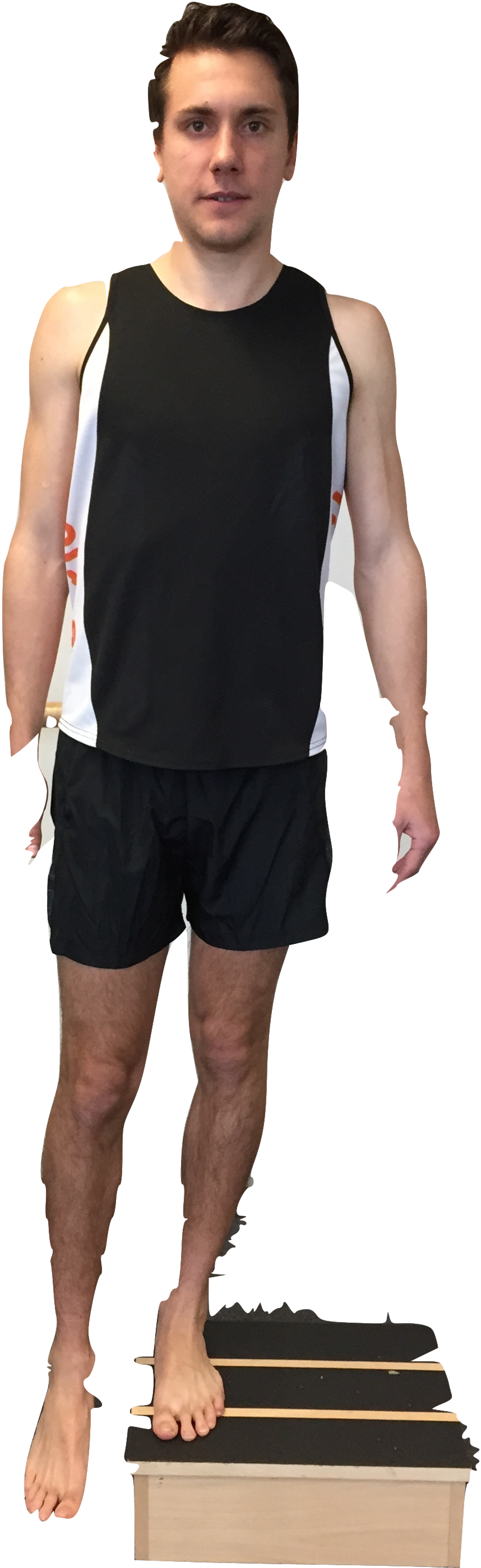 | Participant in standing on operated leg with other foot level but not on a step, holding rail for support if necessary. | Pelvis lowers on non-operated side so that foot is below the step level without flexion of the supporting limb. | 1 sets of 10 repetitions | Gravity only | Participant should start to fatigue at rep no. 7 as measured by Borg scale of exertion level 7. | Completion with minimal fatigue, with no significant increase in knee pain and no lateral hip pain on the operated leg. | Borg scale < 5, pain < 5/10 (knee), pain=0 (lateral hip). | 3 sets of 10 repetitions | Gravity only |

Reps, repetitions; rpm, revolutions per minute; Borg Scale, (0-10 scale).
